# Structural and Thermal Properties of Ethylene-Norbornene Copolymers Obtained Using Vanadium Homogeneous and SIL Catalysts

**DOI:** 10.3390/polym12112433

**Published:** 2020-10-22

**Authors:** Paweł Groch, Anna Bihun-Kisiel, Aleksandra Piontek, Wioletta Ochędzan-Siodłak

**Affiliations:** Faculty of Chemistry, Opole University, Oleska 48, 45-052 Opole, Poland; abihun@uni.opole.pl (A.B.-K.); aolszowy@uni.opole.pl (A.P.); wsiodlak@uni.opole.pl (W.O.-S.)

**Keywords:** ethylene, norbornene, copolymerization, post-metallocene catalyst, NMR investigation, DSC and SSA investigation

## Abstract

The series of ethylene-norbornene (E-NB) copolymers was obtained using different vanadium homogeneous and supported ionic liquid (SIL) catalyst systems. The ^13^C and ^1^H NMR (carbon and proton nuclear magnetic resonance spectroscopy) together with differential scanning calorimetry (DSC) were applied to determine the composition of copolymers such as comonomer incorporation (*C_NB_*), monomer dispersity (*MD*), monomer reactivity ratio (*r_e_*), sequence length of ethylene (*l_e_)* and tetrad microblock distributions. The relation between the type of catalyst, reaction conditions and on the other hand, the copolymer microstructure, chain termination reaction analyzed by the type of unsaturation are discussed. In addition, the thermal properties of E-NB copolymers such as the melting and crystallization behavior, like also the heterogeneity of composition described by successive the self-nucleation and annealing (SSA) and the dispersity index (DI) were determined.

## 1. Introduction

Cyclic olefins copolymers (COCs) have gained attention owing to their unique properties, which make them attractive as high-tech engineering plastics. The most versatile and interesting COCs are those of ethylene (E) with norbornene (NB) [1,2]. The COCs produced by copolymerization of ethylene with norbornene are one of few examples of copolymers industrially produced using metallocene catalysts with trade names TOPAS (by TOPAS Advanced Polymers GmbH) [3,4] and APEL (by Mitsui Chemicals, Inc.) [5]. Poly(ethylene-co-norbornene) materials show many interesting properties like a moisture barrier (4–5 times higher than low density polyethylene (LDPE)) [3], good solvent resistance, facile processability, high thermal stability, transparency and stiffness [6]. These copolymers are a promising class of thermoplastics, ranging from highly crystalline solids to elastomers, whose properties depend on the norbornene content and copolymer microstructure [7,8,9,10].

The main characteristics of these materials are directly linked to cyclic comonomer content, sequence distribution (isolated ENE units, alternating NEN units or presence of NN dyads and NNN triads), as well as a stereochemical arrangement of norbornene units. It is important to determine the microstructure of copolymers in order to understand their different polymer properties, so that to improve significantly their commercial use [6,11].

Nuclear magnetic resonance (NMR) spectroscopy is an extremely useful tool for understanding the structure of polymer. Especially the ^13^C NMR is necessary to describe polymer microstructures. In recent years, due to significant development in NMR instrumentation, progress made in calculation methods and in Quantum Mechanical theory has been observed. This helps more accurate prediction of NMR resonance frequencies and thus characterization of the microstructure of polymers. One of the methods of description of the E-NB copolymers microstructure is employing a computer optimization routine, which allows the best fit of the microstructural analysis by ^13^C NMR spectra and the derivation of reactivity ratios for both first and second-order Markov models. It should be emphasized that the spectra of cyclic olefins are relatively complex due to the presence of stereogenic carbon atoms in the polymer chain per monomer unit. Furthermore, chemical shifts of these copolymers do not obey simple additive rules [1,12,13].

Copolymer microstructure and properties are tightly connected with conditions of reaction and type of catalyst. Nowadays, interest in vanadium complexes is currently growing because they enable to obtain polymers of high molecular weight (*M_w_*) and narrow molecular weight distribution (*M_w_/M_n_*), copolymers of ethylene with higher 1-olefins or cyclic olefins with high comonomer incorporation in the copolymer chain [1,14,15,16,17,18,19,20,21,22]. However, a big disadvantage of vanadium compounds is their instability, they are easily reduced to lower, inactive oxidation state. To prevent this, ethyl trichloroacetate (ETA) reactivator is often used. Another method of stabilizing the vanadium active center is applying ligands with electron-donating atoms, oxygen or nitrogen [14,23,24,25,26,27,28,29,30,31,32,33,34,35]. In our previous works it was found that the vanadium metallocene and post-metallocene catalysts with oxazoline ligand activated by the traditional organoaluminium compound (AlEt_2_Cl) and ETA can be successfully used for COC synthesis [35,36,37,38,39,40,41].

In order to increase the stability of catalysts in industry, metal complexes are immobilized on a solid support. Simultaneously, it allows controlling polymer microstructure and morphology as well as eliminating reactor fouling. An extremely interesting way to make heterogeneous catalyst is to use so-called supported ionic liquid (SIL) systems. In the SIL system a thin layer of ionic liquid with dissolved catalyst is anchored on a solid, porous, inorganic or organic support [21,42,43,44,45]. The SIL systems combine the advantages of homogeneous and heterogeneous catalysis, while reducing their disadvantages [42,43]. Moreover, our group showed that the SIL systems were active both in ethylene polymerization and copolymerization of ethylene with α-olefins and cyclic olefins [21,44,46].

This work presents structural and physicochemical analysis of ethylene-norbornene copolymers obtained using homogeneous or heterogeneous (SIL) vanadium metallocene and post-metallocene catalysts. The microstructures of these copolymers were characterized in detail by ^13^C NMR and ^1^H NMR. The structural properties and compositions of copolymers were analyzed and discussed on the basis of the obtained results with use for example Finneman-Ross and Randall method. It should be noted that in our best knowledge there is no study related to thermal properties of E-NB copolymers due to their usually has an amorphous form. This study was carried out, by means of differential scanning calorimetry (DSC) on obtained polymeric products. In addition, the successive self-nucleation and annealing (SSA) allowed to determine the lamellar thickness, dispersity index and fraction share of obtained E-NB copolymers which are significantly important parameters of semi-crystalline polymeric products.

## 2. Materials and Methods

### 2.1. Compounds

**Figure 1:**
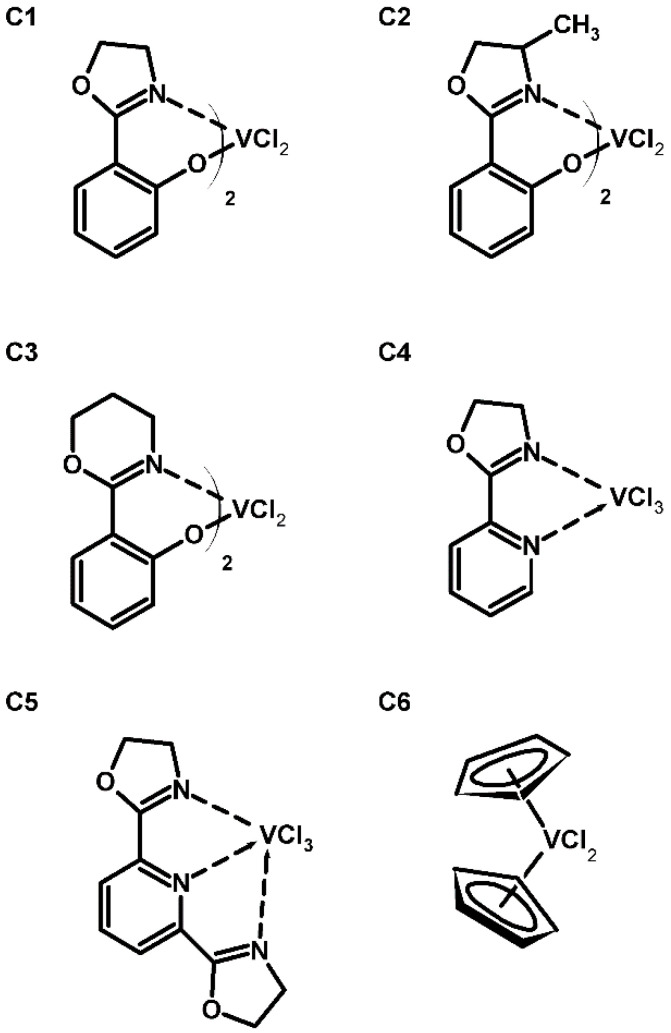
Structures of the vanadium complexes applied for E-NB copolymerization.

**Figure 2:**
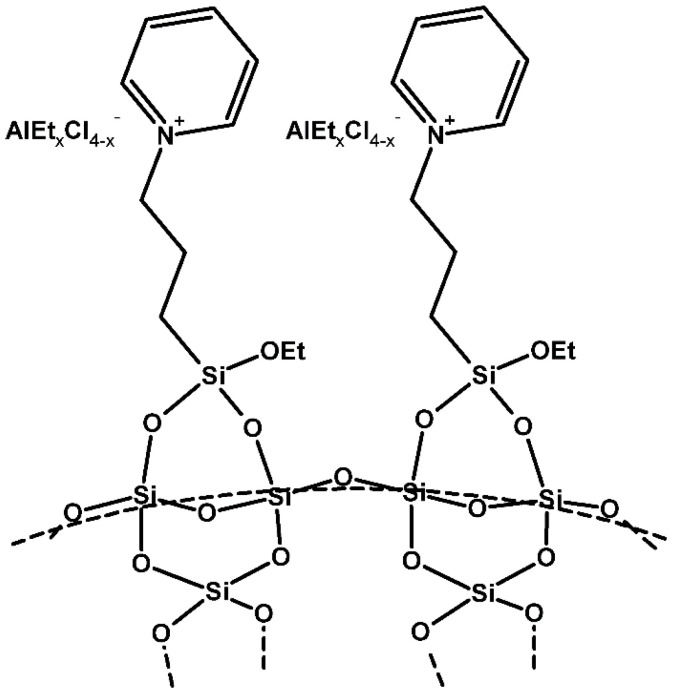
Schematic presentation of silica covered by the ionic liquid.

The E-NB copolymers were prepared according to the procedures previously published [21,26,44,45,46]. The vanadium complexes with the following ligands were applied for the copolymerization reaction: 2-(4,5-dihydro-1,3-oxazolin-2-yl)phenol (**C1**), 2-(4-methyl-4,5-dihydro-1,3-oxazolin-2-yl)phenol (**C2**), 2-(5,6-dihydro-4H-1,3-oxazin-2-yl)phenol (**C3**), 2-(1,3-oxazolin-2-yl)pyridine (**C4**), 2,6-bis(1,3-oxazolin-2-yl)pyridine (**C5**) and cyclopentadienyl (**C6**) (Figure 1). Additionally, the **C4**, **C5** and **C6** catalysts were immobilized on the silica covered by 1-[3-(triethoxysilyl)propyl]pyridinium ethylchloroaluminate ionic liquid (Figure 2), which results in supported ionic liquid (SIL) catalyst, respectively **SIL/C4**, **SIL/C5** and **SIL/C6**. The norbornene incorporation in the copolymer chain (*C_NB_*) fell within the range of 4.3–34.7 mol%, as determined by the ^13^C NMR method. Molecular weight (*M_w_*) of E-NB copolymers evaluated on an Alliance 135 GPCV 2000 apparatus was in the range of 90–1138 × 10^3^ g/mol. Neat polyethylenes obtained under the same conditions were used as reference material.

### 2.2. Characterization Techniques

NMR experiments were performed in standard 5 mm NMR tubes at 120 °C in 1,2-dichlorobenzene-d4 using a UltraShield Bruker spectrometer (400 MHz) equipped with a smart probe. ^1^H NMR spectra were referenced to the residual protons of the non-deuterated solvent used. ^13^C NMR spectra were referenced internally to the ^13^C resonances of the NMR solvent. The norbornene content in copolymers was calculated according to the formula [2*I*(**C7**) + *I*(**C1**–**C2**) + *I*(**C2**–**C3**)] × 100/3*I*(CH_2_), where *I*(CH_2_), *I*(**C7**), *I*(**C1**–**C2**) and *I*(**C2**–**C3**) are the peak areas in the ranges 26–30, 30–36, 34–42 and 43–54 ppm of ^13^C NMR spectra [6,47].

Differential scanning calorimetry (DSC) scans were carried out on a DSC1 Mettler Toledo instrument. The sample in an amount of 5 mg was placed in an aluminum pan and the measurement was carried out using a heating and cooling rate of 10 °C min^−1^ at nitrogen atmosphere with the gas flow 30 mL min^−1^. In order to remove the thermal history of samples the melting temperature (*T*_m_) and heat of melting (*ΔH*_m_) values were recorded during the second heating. The successive self-nucleation and annealing (SSA) investigation was performed by DSC following the procedure previously published [48] at nitrogen atmosphere with the gas flow of 30 mL min^−1^. The samples were heated up to 170 °C and maintained at this level for 20 min and then they were cooled down to 0 °C at the rate of 5 °C min^−1^. This cycling procedure was repeated several times for temperatures from 140 to 67 °C at 3 °C intervals [48]. The lamellar thickness for polymeric samples was determined by deconvolution of broad signal in DSC curve and the Gibbs-Thomson Equation (1) [49]:

(1) $$T_{m}=T_{m}^{o}\left(\frac{1-2\sigma_{e}}{\Delta H_{f}\times L_{GT}}\right)$$

The following parametric values were used in calculations [19,20,21]: $T_{m}^{o}$ equilibrium melting temperature of a perfect crystal is 145.5 °C, Δ*H_f_* is related to heat of fusion per unit volume for a perfect crystal is 290 J cm^−3^ and σ_e_ is crystallite specific surface free energy fell within the range 90 mJ m^−2^. In addition, the *DI* values were calculated by the ratio of the melting enthalpy of each peak to the maximum melting enthalpy in the SSA curves of the copolymer sample.

## 3. Results

### 3.1. Copolymers Composition

Table 1 presents the selected parameters of the copolymerization reaction, including the type of catalyst, as well as the selected parameters of the obtained copolymers. The increase of norbornene concentration in the reaction feed results, as expected, in an increase of norbornene content (*C_NB_*) incorporated into polymer chain, irrespective of the kind of catalyst complex used. It should be noted that despite the relatively low range of NB concentration studied, from 0.1 to 1.5 mol/dm^3^, the determined *C_NB_* values range from 4 up to 35 mol%. At the highest studied NB concentration in the feed (1.5 mol/dm^3^), the *C_NB_* values vary in the range 27–35 mol%. Some correlation between the catalyst structure and the *C_NB_* values can be observed. In the series of the catalysts with phenolate ligands the norbornene incorporation is higher for **C2** and **C3** than for **C1** analogue. Similarly, the norbornene incorporation is higher for the catalyst with bidentate **C4** than tridentate **C5** pyridinium ligands. It should be noticed higher *C_NB_* values for **C4** as compared to **C1** catalyst with the same oxazoline substituent. High NB incorporation was also obtained using vanadocene catalyst (**C6**). In case of the SIL supported catalyst systems the presented results indicate that they produce the copolymer with higher NB content in comparison with homogeneous analogues, regardless of the type of catalyst.

The copolymers are characterized by molecular weight (*M_w_*) in the range from 90 × 10^3^ to 1100 × 10^3^ g/mol, which decrease with increasing content of NB, like also by high homogeneity (*M_w_/M_n_* about 2). The copolymers produced using the SIL catalysts show generally higher molecular weights (especially at the extreme content in the reaction feed) in comparison to the copolymers obtained over non-supported analogues (Table 1).

The influence of structure of complexes on incorporation ability as well as the difference between the non-supported and SIL catalyst systems is shown by the reactivity ratios of ethylene (*r_e_*) calculated using the Fineman-Ross method [50] (Table S1 in the supplementary file and Table 1). The Fineman-Ross method was successfully used in the work related to the copolymerization of ethylene with norbornene [51].

In case of the E-NB copolymerization over homogeneous catalytic complexes, the *r_e_* values increase as follows: **C6** (6.5) > **C4** (7.2) > **C5** (7.9) > **C1** (8.6) > **C3** (10.8) > **C2** (12.2) (Table 1, items 1–12, 16–18, 22–24). It should be noted that the presence of methyl substituent in oxazoline ligands in **C2** complex results in increase of *r_e_* value (decrease of norbornene reactivity) in comparison with **C1** complex. This result is associated with an increase in electron density in ligand and hence stronger interaction of ligand with metal center what affects increase of steric hindrance. In turn, a slightly larger oxazine substituents in **C3** leads to higher steric hindrance in comparison with oxazoline substituents in **C1** and increase of the value *r_e_*. Interestingly, despite the fact that the **C4** pyridinium complex is characterized by less steric hindrance than **C1** complex, the value of *r_e_* is markedly lower. In addition, the tridentate **C5** pyridine complex with larger ligand which surrounded the metal center, exhibits only slightly higher *r_e_* value in comparison with bidentate **C4** pyridine complex.

The comparison of the catalysts systems **C4**, **SIL/C4**, **C5** and **SIL/C5**, **C6** and **SIL/C6** (Table 1, items 10–15, 16–21 and 22–27) shows lower values of *r_e_* for the SIL-supported catalysts than non-supported analogues. The highest influence of immobilization process on the reactivity of norbornene is observed in the catalyst **SIL/C4** (Table 1, items 10–15). This shows that the immobilization of complexes on support generally causes an increase of reactivity ratio of norbornene.

In order to determine the differences between the microstructure of the analyzed copolymers, the structural parameters characterizing the copolymer chain were calculated using the Randall method [52]. As expected, the number-average sequence length of ethylene (*l_e_*) decreases with the increase of NB concentration in the feed, which shows that shorter ethylene sequences are created. Regardless of the type of catalyst, there is clear correlation between norbornene content (*C_NB_*) in the copolymer and the value of *l_e_*, which decreases with the increasing ability of the catalytic system to incorporate norbornene in the polymer chain. Again, the SIL supported catalyst reveal lower values of *l_e_*, which indicates shorter length of ethylene sequences as compared to non-supported analogues.

The calculated values of monomer dispersity (*MD*) for the E-NB copolymers also depends on the norbornene concentration and incorporation. For low NB incorporation the *MD* values are in the range of 96–80, which indicates that all or almost all norbornene units in the macromolecules are isolated (Table 1, items, 1, 4, 7, 10, 13, 16, 19, 22 and 25, Figures S1–S27 in the supplementary file). Decrease of *MD* values indicates higher NB incorporation and the presence of different kind of norbornene sequence.

**Figure 3:**
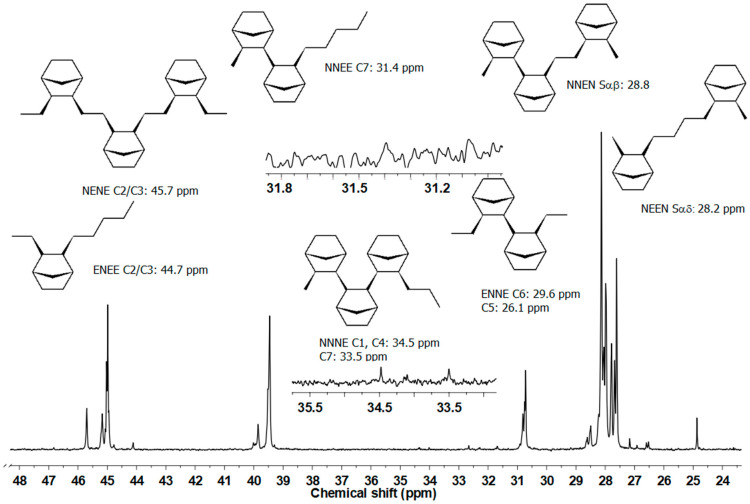
^13^C nuclear magnetic resonance (NMR) spectra of E-NB copolymer synthesized by SIL/C5 catalytic system.

The type of sequences was evaluated by the E-NB copolymer tetrad distributions using ^13^C NMR method (Figure 3).

The results were obtained only for copolymers with the lowest *MD* values (from 64.7 to 72.8) synthesized over homogeneous and SIL catalyst systems (Table 2). It should be noted that in all the studied copolymers the NNNN tetrads were not observed. The NNNE tetrads did not appear or appear in small amount in the copolymers obtained using homogenous catalyst (**C2**–**C4**), despite to very high norbornene content. Immobilization of complexes on support generally results in much higher content of NENE, NNEE, ENNE as well as NNNE tetrads (alternating E/N units and norbornene blocks, respectively). Moreover, immobilization process causes decrease of isolated ENEE tetrads (Table 2, items 1–21 and 24, 27).

### 3.2. Unsaturation Group Study of E-NB Copolymers

**Figure 4:**
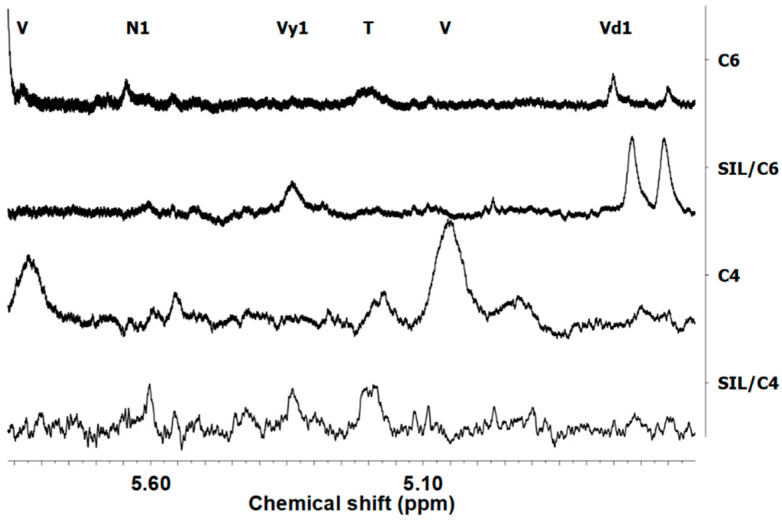
^1^H NMR spectra of E-NB copolymers obtained using homogeneous (**C6**, **C4**) and heterogeneous (**SIL/C6**, **SIL/C4**) catalyst with the highest NB incorporation degree (Table 1, item 27, 24, 12, 15).

The catalytic systems have an influence on the chain termination reaction in the E-NB copolymerization as it is shown by determination of the type of unsaturated groups for the products obtained using the ^1^H NMR (Figure 4).

The ^1^H NMR study was performed for the E-NB copolymers obtained by the selected homogeneous and heterogeneous catalyst (**C4**, **SIL/C4**), in which a considerable influence of immobilization on the value of *r_e_* was observed, as well as the metallocene (**C6**, **SIL/C6**), for comparison of both types of complexes. The spectra of E-NB copolymers show the presence of a large number of peaks related to different kind of unsaturation groups, which indicates different way of termination reactions in copolymerization.

All resonances presented in the ^1^H NMR spectra were assigned by comparison with the literature data [53]. The multiples within the range of 5.9–5.7 and 5.0–4.9 ppm are assigned to vinyl groups (CH_2_=CHR; V), which appears due to β-H elimination after ethylene insertion. The signal at 5.6 ppm is characteristic for norbornenyl end group structure. The signal at 5.3 ppm is assigned to the vinylene unsaturated groups after 2,1-insertion (RCH=CHR’; Vy1). It should be noted that the signal at 5.2 ppm is also observed, which corresponds to the presence of trisubstituted vinylene groups (RR’C=CHR”; T). This kind of unsaturation is formed when the comonomer is inserted as the last monomer and this is followed by isomerization and β-H transfer to the metal center [53]. In addition, two peaks at 4.73 ppm and 4.60 ppm correspond to the geminal protons of two different vinylidene structures (VD1 and VD2, internal and external unsaturated groups, respectively).

It should be noted that in case of **C6** and **C6/SIL** metallocene complexes the termination reaction occurs in an unusual way. The metallocene **C6** and **SIL/C6** do not prefer the termination reaction, followed by the β-H elimination after the ethylene insertion, which is shown by the absence of vinyl groups (CH_2_=CHR; V) in the range of 5.9–5.7 and 5.0–4.9 ppm (Figure 4). These results are unusual for the polymerization preformed using the metallocene catalytic systems, in which the β-H elimination is dominant termination reaction [54]. This phenomenon can be explained by the influence of alkylaluminium activator on termination reaction, what was previously observed in our work [55]. The dominant unsaturated groups in the E-NB copolymerization catalyzed by the homogeneous **C6** complex were trisubstituted vinylene like also vinylidene groups (T, VD1 and VD2). However, the immobilization of metallocene complex results in disappearance of the trisubstituted vinylene groups in the copolymer and appearance of vinylene groups. These results clearly indicate that the ionic liquid influences both the suppression of isomerization reaction and the β-H transfer to the metal center of the last monomer and favors the comonomer 2,1-type insertion. It should be noted that the ionic liquid significant changes of the catalytic mechanism of termination polymer chain of metallocene complex.

The E-NB copolymers obtained by the homogeneous catalyst **C4** are characterized by the presence of trisubstituted vinylene groups in contrast to metallocene **C6** and **SIL/C6** complexes. This result indicates that the alkylaluminium activator does not influence on mechanism of termination reaction in copolymerization. In turn, the immobilization of postmetallocene complex leads to suppression of β-H elimination reaction, which is associated with the change of pathway of termination reaction excluding influence of activator (Figure 4). In addition, the observed presence of the vinylene groups in copolymers indicates that the ionic liquid causes the 2,1-type of insertion of comonomer.

### 3.3. Thermal Properties of Copolymers

**Figure 5:**
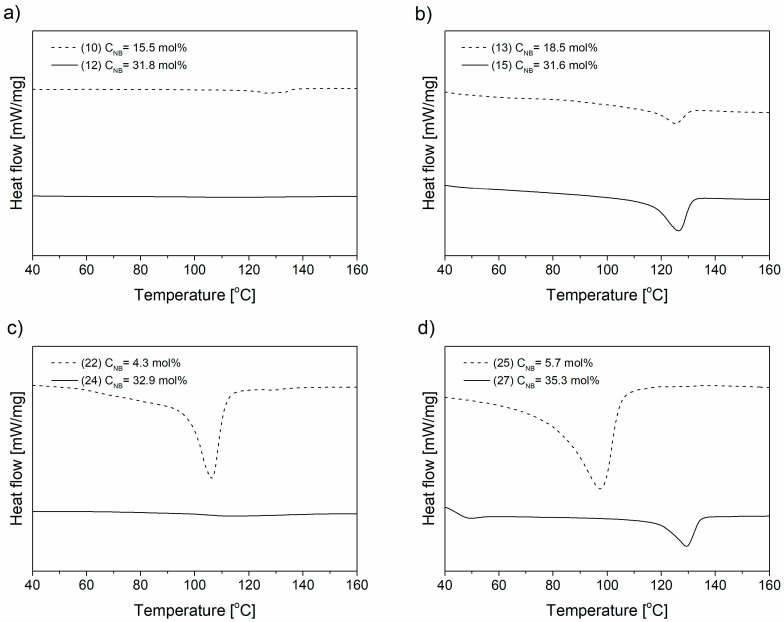
The endothermic curves of E-NB copolymers obtained by **C4** (**a**), **SIL/C4** (**b**), **C6** (**c**), **SIL/C6** (**d**) complex (items according to Table 1).

The kind of catalytic system and degree of norbornene incorporation influence on the melting and crystallization behavior of the E-NB copolymers as it is shown using DSC method (Figure 5 and Figures S28–S32 in the Supplementary File).

The E-NB copolymers obtained over the homogeneous catalytic systems with the highest comonomer degree were amorphous (Figure 5a,c and Figures S28–S31 in the Supplementary File). The similar results are observed in case of ethylene copolymerization with norbornene over homogeneous metallocene catalytic systems [6]. Noteworthy is that the E-NB copolymers synthesized using the immobilized catalytic systems (**SIL/C4**, **SIL/C5** and **SIL/C6**) are characterized by generally increased intensity of melting peak, what is atypical behavior of the E-NB copolymers (Figure 5b,d and Figure S32 in the Supplementary File). This phenomenon can be explained by packing of the growing polymer chain in the nonpolar part of ionic liquid in catalytic system, which in turn leads to markedly increased crystalline structure.

For the copolymers synthesized over the catalysts SIL/C4, SIL/C5 and SIL/C6, the endothermic peak in the DSC thermograms became broader with the increase of NB content (Figure 5b,d and Figure S32 in the Supplementary File), what suggests the increase copolymers heterogeneity.

The NB incorporated into polymer chain causes not only the increase of crystalline structure but also increase of the heterogeneity of composition of copolymer chain in comparison with neat PE.

**Figure 6:**
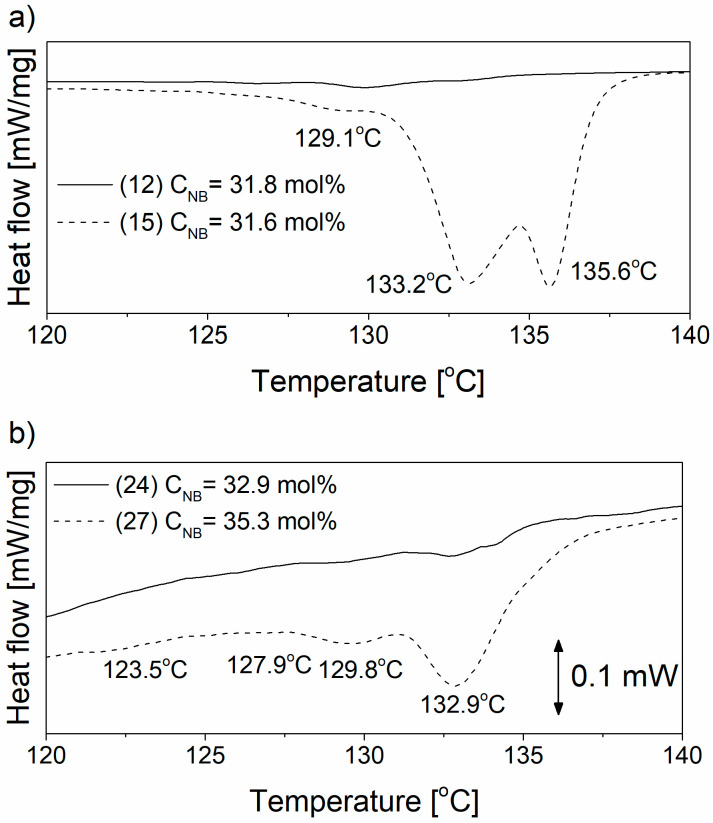
Successive self-nucleation and annealing (SSA) curves of E-NB copolymers obtained by catalysts (**a**) **C4**, **SIL/C4** and (**b**) **C6**, **SIL/C6** (items according to Table 1).

Therefore, the successive self-nucleation and annealing (SSA) analyses were performed by DSC, which is a commonly used thermal fractionation technique [48,56]. The SSA were performed for the selected copolymers synthesized over homogeneous catalyst **C4**, **C6** and immobilized counterparts **SIL/C4**, **SIL/C6** at the highest NB content (Table 1, items 12, 24 and 15, 27). The multiple melting peaks were observed in DSC curves for all of the analyzed copolymer samples but some differences in the number of peaks as well as in position of their maxima and in intensity were observed (Figure 6).

The E-NB copolymer synthesized by the catalyst **SIL/C4** with 31.6 wt% of NB is characterized by three melting peaks at 129.1, 133.2 and 135.6 °C. It is interesting to note that the melting peak at 135.6 °C is very similar to that of neat polyethylene and this at 136.3 °C can be associated with the presence of polyethylene sequence in the copolymer chain with a significantly low NB content. In turn, for the copolymer produced by the catalyst **SIL/C6**, four separated peaks, at 123.5, 127.9, 129.8 and 132.9 °C, are observed in the thermogram. The number of peaks shows that the composition of this copolymer is much more heterogeneous, in contrast to that obtained using the catalyst **SIL/C4** (Figure 6). The fraction with the lowest lamellar thicknesses can be assigned to the presence of NNNE tetrads, which is confirmed by ^13^C NMR (Table 2). In addition, the dispersity index (DI) was determined in order to evaluate the heterogeneity of E-NB copolymers (Table 3).

**Figure 7:**
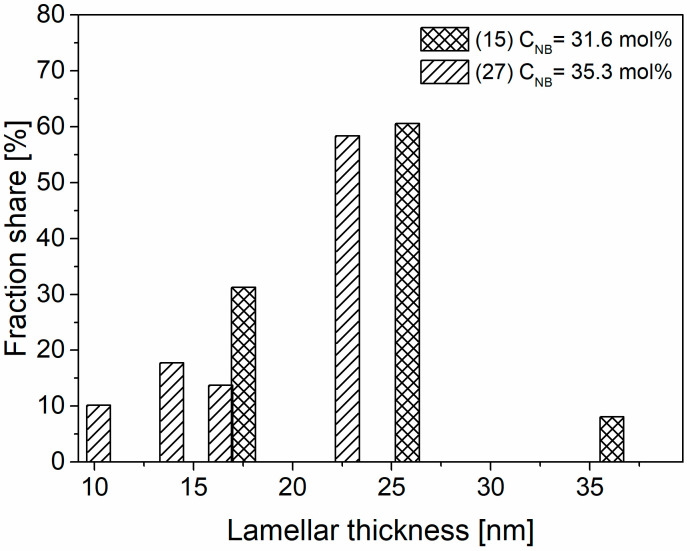
Distribution of lamellar thickness for E-NB copolymers obtained using the catalysts **SIL/C4** and **SIL/C6** (items according to Table 1).

The *DI* values indicates that the E-NB copolymer synthesized using the catalyst **SIL/C4** is more homogeneous in comparison with polymer obtained using the metallocene analogue **SIL/C6**, despite the similar NB content. It should be explained that the E-NB copolymer produced using the catalyst **SIL/C4** is characterized by a constant separation of ethylene sequence in copolymer chain, what causes an appearance of endothermic DSC peaks due to segregation of a constant crystallisable sequence length. Longer segments recrystallized in longer lamellar crystals melt at higher temperatures. The relative number of lamellas having different thicknesses were determined based on the values of *ΔH_m_* for each peak in the SSA thermograms (Figure 7). The catalyst **SIL/C4** produces the E-NB copolymer with higher lamella thickness in comparison with the metallocene analogue **SIL/C6**.

## 4. Conclusions

The presented study indicates that the microstructure and thermal properties of the ethylene-norbornene copolymers produced using vanadium catalysts depend on the type of catalyst used. Both the homogeneous catalyst systems (**C1**–**C6**) and their heterogeneous silica supported ionic liquid analogues (**SIL/C4**–**C6**) were applied. The homogenous catalysts produce the copolymers, which differ in the structure and properties. This seem to be associated with formation sterically more open active pocket of the catalyst center for coordination of the bulky NB comonomer, connected with ligand distortion (**C1**–**C3**) or denticity (**C4**, **C5**), which is further evidenced by high NB incorporation obtained using vanadocene catalyst (**C6**). However, these differences are moderate, in particular, at higher initial NB concentration in the feed. The differences between the homogeneous and supported ionic liquid (SIL) catalysts can be seen, which can be explained by specific interaction of the ionic liquid with the catalyst system. The ionic liquid environment associates the ionic catalyst center, in particular, its anionic counterpart. These results in the formation of weaker ion pair, which enables better access to active center. The nano-segregated structure of ionic liquids should also be considered. The polar ionic liquid phase imposes more tightly packing of the growing non-polar copolymer chain, which results in higher copolymer crystallinity.

The immobilization of catalyst systems generally results in higher reactivity of norbornene in copolymerization with ethylene and in a consequence, in higher norbornene content in the copolymers. In addition, the E-NB copolymers produced using supported catalysts are characterized by higher content of alternating and norbornene blocks and lower content of isolated blocks in comparison with the homogeneous analogues. The chain termination reaction also depends on the kind of catalytic system. The immobilization of complexes significantly influences the way of chain termination reaction in comparison with homogeneous analogue.

The thermal properties of copolymers strongly depend on the kind of catalytic system. The E-NB copolymers obtained by the homogeneous catalytic system have amorphous form in contrast to the products synthesized by the supported SIL catalysts, which are characterized by semi-crystalline structure. The growing polymer chain is located in the limited space of nonpolar part of the ionic liquid, which results in formation of crystalline structure. In addition, the SSA analysis of E-NB copolymers obtained by the supported SIL catalyst with similar NB content reveals that higher homogeneity and lamellar thickness have copolymers produced using the postmetallocene catalyst.

## Figures and Tables

**Table 1:** Selected parameters of E-NB copolymers and copolymerization reaction performed using studied vanadium catalysts.

Item	Catalyst	NB Feed [mol/dm^3^]	*C_NB_* [mol%]	*M_w_* × 10^−3^ [g/mol]	*M_w_/M_n_*	*r_e_*	*l_e_*	*MD*
1	**C1**	0.5	12.3	260	1.6	8.6	8.1	87.7
2		1.0	22.1	200	1.8		4.5	77.9
3		1.5	27.5	180	1.9		3.6	72.5
4	**C2**	0.5	17.1	160	1.7	12.2	5.8	82.9
5		1.0	20.7	130	1.9		4.8	79.3
6		1.5	27.2	90	2.2		3.7	72.8
7	**C3**	0.5	19.4	190	1.7	10.8	5.2	80.6
8		1.0	26.5	170	1.7		3.8	73.5
9		1.5	30.1	160	1.9		3.3	69.9
10	**C4**	0.5	15.5	679	1.9	7.2	6.5	84.5
11		1.0	23.0	420	1.9		4.3	77.0
12		1.5	31.8	187	2.0		3.1	68.2
13	**SIL/C4**	0.5	18.5	852	2.0	8.7	5.4	81.5
14		1.0	25.2	461	2.3		4.0	74.8
15		1.5	31.6	214	2.5		3.2	68.4
16	**C5**	0.5	14.4	679	1.9	7.9	6.9	85.6
17		1.0	21.9	420	1.9		4.6	78.1
18		1.5	30.0	178	2.0		3.3	69.6
19	**SIL/C5**	0.5	18.9	679	1.9	6.9	5.3	81.1
20		1.0	26.8	420	1.9		3.7	73.2
21		1.5	34.7	198	2.0		2.9	65.3
22	**C6**	0.1	4.3	1047	2.5	6.5	23.3	95.7
23		1.0	23.6	470	2.9		4.2	76.4
24		1.5	32.9	157	2.0		3.0	67.1
25	**SIL/C6**	0.1	5.7	1138	1.5	6.0	17.5	94.3
26		1.0	26.1	265	2.9		3.8	73.9
27		1.5	35.3	225	3.0		2.8	64.7

*C_NB_*—determined by ^13^C NMR, *M_w_* and *M_w_/M_n_* obtained by GPC, *l_e_*—number-average sequence length of ethylene, *r_e_*—reactivity ratio, *MD*—monomer dispersity.

**Table 2:** Tetrad distributions of E-NB copolymers obtained over different homogeneous and SIL catalyst systems.

^a^ Item	Catalyst	^b^*C_NB_* [mol%]	^b^ Tetrad									
			EEEE	NEEE	NEEN	ENEE	NNEE	NENE	NNEN	ENNE	NNNE	NNNN
1	**C1**	27.5	0.651	0.068	0.045	0.110	0.004	0.107	0.005	0.007	0.005	0.000
6	**C2**	27.2	0.738	0.049	0.029	0.104	0.000	0.064	0.015	0.001	0.000	0.000
9	**C3**	30.1	0.587	0.073	0.062	0.117	0.000	0.126	0.035	0.000	0.000	0.000
12	**C4**	31.8	0.651	0.072	0.059	0.109	0.003	0.104	0.000	0.002	0.000	0.000
15	**SIL/C4**	31.6	0.613	0.074	0.056	0.093	0.006	0.130	0.004	0.012	0.012	0.000
18	**C5**	30.0	0.669	0.078	0.062	0.031	0.007	0.125	0.011	0.008	0.009	0.000
21	**SIL/C5**	34.7	0.680	0.084	0.058	0.014	0.003	0.133	0.005	0.003	0.020	0.000
24	**C6**	32.9	0.666	0.067	0.040	0.112	0.002	0.107	0.000	0.002	0.004	0.000
27	**SIL/C6**	35.3	0.620	0.071	0.055	0.110	0.007	0.120	0.000	0.009	0.008	0.000

^a^ Item according to Table 1, NB concentration in reaction feed: 1.5 mol/dm^3^, ^b^ determined by ^13^C NMR.

**Table 3:** *DI* values for E-NB copolymers obtained over SIL catalysts.

Item	Catalyst	^a^*C_NB_* [mol%]	*DI*			
			F1	F2	F3	F4
15	**SIL/C4**	31.6	0.52	1.00	0.13	-
27	**SIL/C6**	35.3	0.17	0.30	0.24	1.00

^a^ determined by ^13^C NMR.

## Supplementary Materials

The following are available online at https://www.mdpi.com/2073-4360/12/11/2433/s1, Figure S1: The ^13^C NMR spectrum of E-NB copolymer with (1) C_NB_ = 12.3 mol% obtained by **C1** complex (item according to Table 1), Figure S2: The ^13^C NMR spectrum of E-NB copolymer with (2) C_NB_ = 22.1 mol% obtained by **C1** complex (item according to Table 1), Figure S3: The ^13^C NMR spectrum of E-NB copolymer with (3) C_NB_ = 27.5 mol% obtained by **C1** complex (item according to Table 1), Figure S4: The ^13^C NMR spectrum of E-NB copolymer with (4) C_NB_ = 17.1 mol% obtained by **C2** complex (item according to Table 1), Figure S5: The ^13^C NMR spectrum of E-NB copolymer with (5) C_NB_ = 20.7 mol% obtained by **C2** complex (item according to Table 1), Figure S6: The ^13^C NMR spectrum of E-NB copolymer with (6) C_NB_ = 27.2 mol% obtained by **C2** complex (item according to Table 1), Figure S7: The ^13^C NMR spectrum of E-NB copolymer with (7) C_NB_ = 19.4 mol% obtained by **C3** complex (item according to Table 1), Figure S8: The ^13^C NMR spectrum of E-NB copolymer with (8) C_NB_ = 26.5 mol% obtained by **C3** complex (item according to Table 1), Figure S9: The ^13^C NMR spectrum of E-NB copolymer with (9) C_NB_ = 30.1 mol% obtained by **C3** complex (item according to Table 1), Figure S10: The ^13^C NMR spectrum of E-NB copolymer with (10) C_NB_ = 15.5 mol% obtained by **C4** complex (item according to Table 1), Figure S11: The ^13^C NMR spectrum of E-NB copolymer with (11) C_NB_ = 23.0 mol% obtained by **C4** complex (item according to Table 1), Figure S12: The ^13^C NMR spectrum of E-NB copolymer with (12) C_NB_ = 31.8 mol% obtained by **C4** complex (item according to Table 1), Figure S13: The ^13^C NMR spectrum of E-NB copolymer with (13) C_NB_ = 18.5 mol% obtained by **SIL/C4** complex (item according to Table 1), Figure S14: The ^13^C NMR spectrum of E-NB copolymer with (14) C_NB_ = 25.2 mol% obtained by **SIL/C4** complex (item according to Table 1), Figure S15: The ^13^C NMR spectrum of E-NB copolymer with (15) C_NB_ = 31.6 mol% obtained by **SIL/C4** complex (item according to Table 1), Figure S16: The ^13^C NMR spectrum of E-NB copolymer with (16) C_NB_ = 14.4 mol% obtained by **C5** complex (item according to Table 1), Figure S17: The ^13^C NMR spectrum of E-NB copolymer with (17) C_NB_ = 21.9 mol% obtained by **C5** complex (item according to Table 1), Figure S18: The ^13^C NMR spectrum of E-NB copolymer with (18) C_NB_ = 30.0 mol% obtained by **C5** complex (item according to Table 1), Figure S19: The ^13^C NMR spectrum of E-NB copolymer with (19) C_NB_ = 18.9 mol% obtained by **SIL/C5** complex (item according to Table 1), Figure S20: The ^13^C NMR spectrum of E-NB copolymer with (20) C_NB_ = 26.8 mol% obtained by **SIL/C5** complex (item according to Table 1), Figure S21: The ^13^C NMR spectrum of E-NB copolymer with (21) C_NB_ = 34.7 mol% obtained by **SIL/C5** complex (item according to Table 1), Figure S22: The ^13^C NMR spectrum of E-NB copolymer with (29) C_NB_ = 4.3 mol% obtained by **C6** complex (item according to Table 1), Figure S23: The ^13^C NMR spectrum of E-NB copolymer with (30) C_NB_ = 23.6 mol% obtained by **C6** complex (item according to Table 1), Figure S24: The ^13^C NMR spectrum of E-NB copolymer with (31) C_NB_ = 32.9 mol% obtained by **C6** complex (item according to Table 1), Figure S25: The ^13^C NMR spectrum of E-NB copolymer with (32) C_NB_ = 5.7 mol% obtained by **SIL/C6** complex (item according to Table 1), Figure S26: The ^13^C NMR spectrum of E-NB copolymer with (33) C_NB_ = 26.1 mol% obtained by **SIL/C6** complex (item according to Table 1), Figure S27: The ^13^C NMR spectrum of E-NB copolymer with (34) C_NB_ = 35.3 mol% obtained by **SIL/C6** complex (item according to Table 1), Figure S28: The endothermic curves of E-NB copolymers obtained by **C1** complex (items according to Table 1), Figure S29: The endothermic curves of E-NB copolymers obtained by **C2** complex (items according to Table 1), Figure S30: The endothermic curves of E-NB copolymers obtained by **C3** complex (items according to Table 1), Figure S31: The endothermic curves of E-NB copolymers obtained by **C5** complex (items according to Table 1), Figure S32: The endothermic curves of E-NB copolymers obtained by **SIL/C5** complex (items according to Table 1). Table S1. Structural parameters characterizing the ethylene/norbornene copolymers.
