# Supplementary material for: Structural and Thermal Properties of Ethylene-Norbornene Copolymers Obtained Using Vanadium Homogeneous and SIL Catalysts
Source: Polymers (Basel). 2020 Oct 22;12(11):2433. doi: 10.3390/polym12112433 (PMC7690451; doi:10.3390/polym12112433)
Supplement: Supplementary file 1 [file polymers-12-02433-s001.pdf]

Supplementary Materials

Structural and thermal properties of ethylene-norbornene copolymers obtained using vanadium homogeneous and SIL catalysts

Paweł Groch*, Anna Bihun-Kisiel, Aleksandra Piontek, Wioletta Ochędzan-Siodłak

Opole University, Faculty of Chemistry, Oleska 48, 45-052 Opole, Poland

*Corresponding author. E-mail address: pawel.groch@uni.opole.pl (P. Groch)

Table of contents

**Figure 1S**	The ^{13}C NMR spectrum of E-NB copolymer with (1) C_{NB}= 12.3 mol% obtained by **C1** complex (item according to Table 1)	3
**Figure 2S**	The ^{13}C NMR spectrum of E-NB copolymer with (2) C_{NB}= 22.1 mol% obtained by **C1** complex (item according to Table 1)	3
**Figure 3S**	The ^{13}C NMR spectrum of E-NB copolymer with (3) C_{NB}= 27.5 mol% obtained by **C1** complex (item according to Table 1)	4
**Figure 4S**	The ^{13}C NMR spectrum of E-NB copolymer with (4) C_{NB}= 17.1 mol% obtained by **C2** complex (item according to Table 1)	4
**Figure 5S**	The ^{13}C NMR spectrum of E-NB copolymer with (5) C_{NB}= 20.7 mol% obtained by **C2** complex (item according to Table 1)	5
**Figure 6S**	The ^{13}C NMR spectrum of E-NB copolymer with (6) C_{NB}= 27.2 mol% obtained by **C2** complex (item according to Table 1)	5
**Figure 7S**	The ^{13}C NMR spectrum of E-NB copolymer with (7) C_{NB}= 19.4 mol% obtained by **C3** complex (item according to Table 1)	6
**Figure 8S**	The ^{13}C NMR spectrum of E-NB copolymer with (8) C_{NB}= 26.5 mol% obtained by **C3** complex (item according to Table 1)	6
**Figure 9S**	The ^{13}C NMR spectrum of E-NB copolymer with (9) C_{NB}= 30.1 mol% obtained by **C3** complex (item according to Table 1)	7
**Figure 10S**	The ^{13}C NMR spectrum of E-NB copolymer with (10) C_{NB}= 15.5 mol% obtained by **C4** complex (item according to Table 1)	7
**Figure 11S**	The ^{13}C NMR spectrum of E-NB copolymer with (11) C_{NB}= 23.0 mol% obtained by **C4** complex (item according to Table 1)	8
**Figure 12S**	The ^{13}C NMR spectrum of E-NB copolymer with (12) C_{NB}= 31.8 mol% obtained by **C4** complex (item according to Table 1)	8
**Figure 13S**	The ^{13}C NMR spectrum of E-NB copolymer with (13) C_{NB}= 18.5 mol% obtained by **SIL/C4** complex (item according to Table 1)	9
**Figure 14S**	The ^{13}C NMR spectrum of E-NB copolymer with (14) C_{NB}= 25.2 mol% obtained by **SIL/C4** complex (item according to Table 1)	9
**Figure 15S**	The ^{13}C NMR spectrum of E-NB copolymer with (15) C_{NB}= 31.6 mol% obtained by **SIL/C4** complex (item according to Table 1)	10
**Figure 16S**	The ^{13}C NMR spectrum of E-NB copolymer with (16) C_{NB}= 14.4 mol% obtained by **C5** complex (item according to Table 1)	10
**Figure 17S**	The ^{13}C NMR spectrum of E-NB copolymer with (17) C_{NB}= 21.9 mol% obtained by **C5** complex (item according to Table 1)	11
**Figure 18S**	The ^{13}C NMR spectrum of E-NB copolymer with (18) C_{NB}= 30.0 mol% obtained by **C5** complex (item according to Table 1)	11
**Figure 19S**	The ^{13}C NMR spectrum of E-NB copolymer with (19) C_{NB}= 18.9 mol% obtained by **SIL/C5** complex (item according to Table 1)	12
**Figure 20S**	The ^{13}C NMR spectrum of E-NB copolymer with (20) C_{NB}= 26.8 mol% obtained by **SIL/C5** complex (item according to Table 1)	12

**Figure 21S**	The ^{13}C NMR spectrum of E-NB copolymer with (21) C_{NB}= 34.7 mol% obtained by **SIL/C5** complex (item according to Table 1)	13
**Figure 22S**	The ^{13}C NMR spectrum of E-NB copolymer with (29) C_{NB}= 4.3 mol% obtained by **C6** complex (item according to Table 1)	13
**Figure 23S**	The ^{13}C NMR spectrum of E-NB copolymer with (30) C_{NB}= 23.6 mol% obtained by **C6** complex (item according to Table 1)	14
**Figure 24S**	The ^{13}C NMR spectrum of E-NB copolymer with (31) C_{NB}= 32.9 mol% obtained by **C6** complex (item according to Table 1)	14
**Figure 25S**	The ^{13}C NMR spectrum of E-NB copolymer with (32) C_{NB}= 5.7 mol% obtained by **SIL/C6** complex (item according to Table 1)	15
**Figure 26S**	The ^{13}C NMR spectrum of E-NB copolymer with (33) C_{NB}= 26.1 mol% obtained by **SIL/C6** complex (item according to Table 1)	15
**Figure 27S**	The ^{13}C NMR spectrum of E-NB copolymer with (34) C_{NB}= 35.3 mol% obtained by **SIL/C6** complex (item according to Table 1)	16
**Figure 28S**	The endothermic curves of E-NB copolymers obtained by **C1** complex (items according to Table 1)	16
**Figure 29S**	The endothermic curves of E-NB copolymers obtained by **C2** complex (items according to Table 1)	17
**Figure 30S**	The endothermic curves of E-NB copolymers obtained by **C3** complex (items according to Table 1)	17
**Figure 31S**	The endothermic curves of E-NB copolymers obtained by **C5** complex (items according to Table 1)	18
**Figure 32S**	The endothermic curves of E-NB copolymers obtained by **SIL/C5** complex (items according to Table 1)	18
**Table 1S**	Structural parameters characterizing the ethylene/norbornene copolymers	19

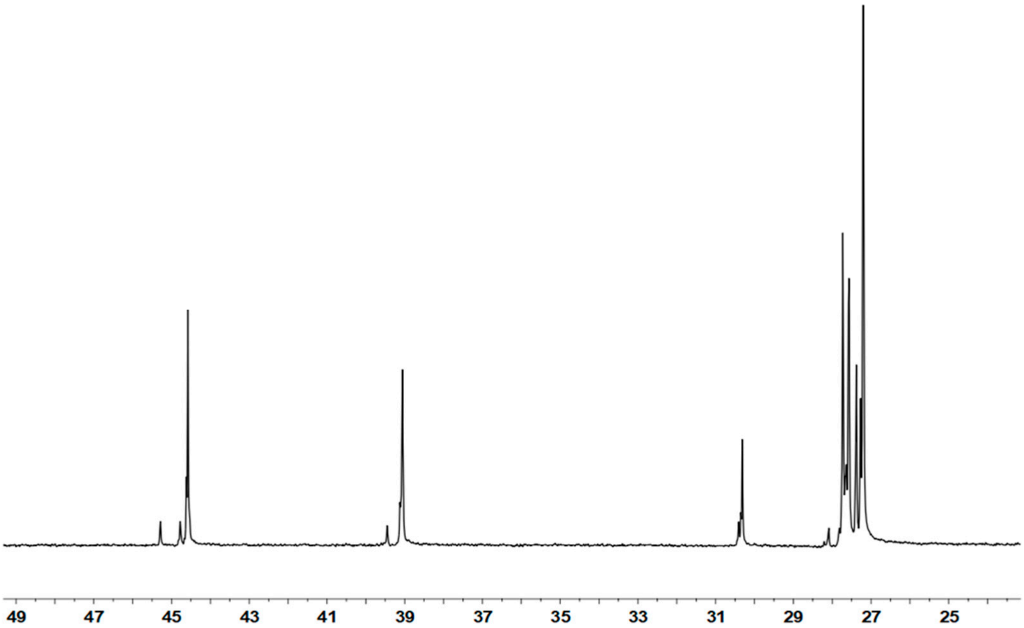

Figure 1S. The ^{13}C NMR spectrum of E-NB copolymer with (1) C_{NB}= 12.3 mol% obtained by **C1** complex (item according to Table 1)

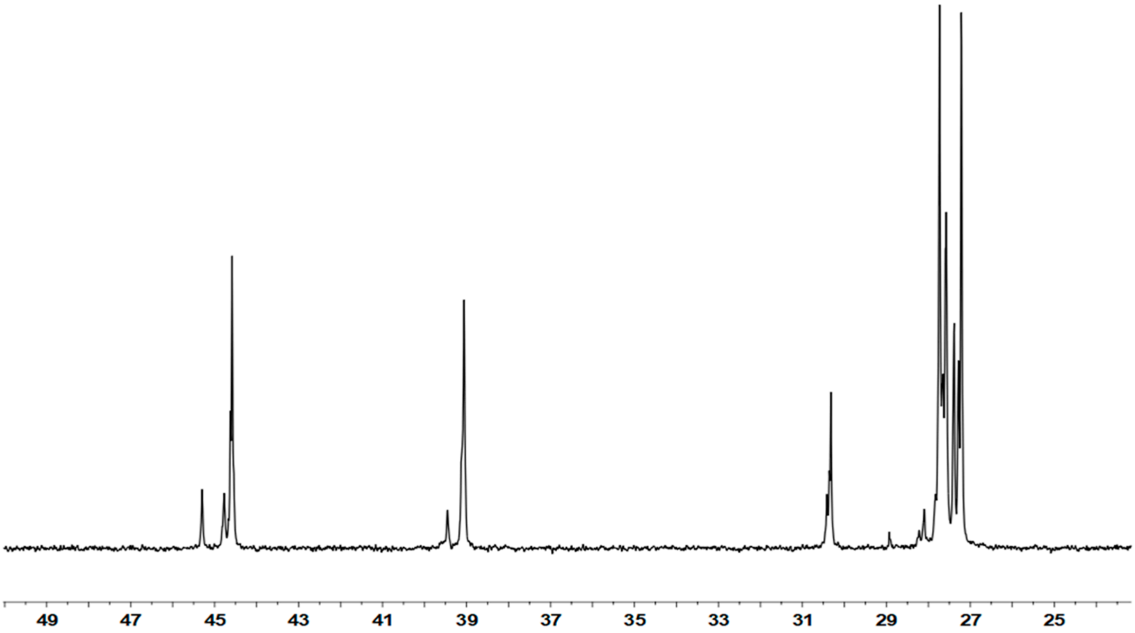

Figure 2S. The ^{13}C NMR spectrum of E-NB copolymer with (2) C_{NB}= 22.1 mol% obtained by **C1** complex (item according to Table 1)

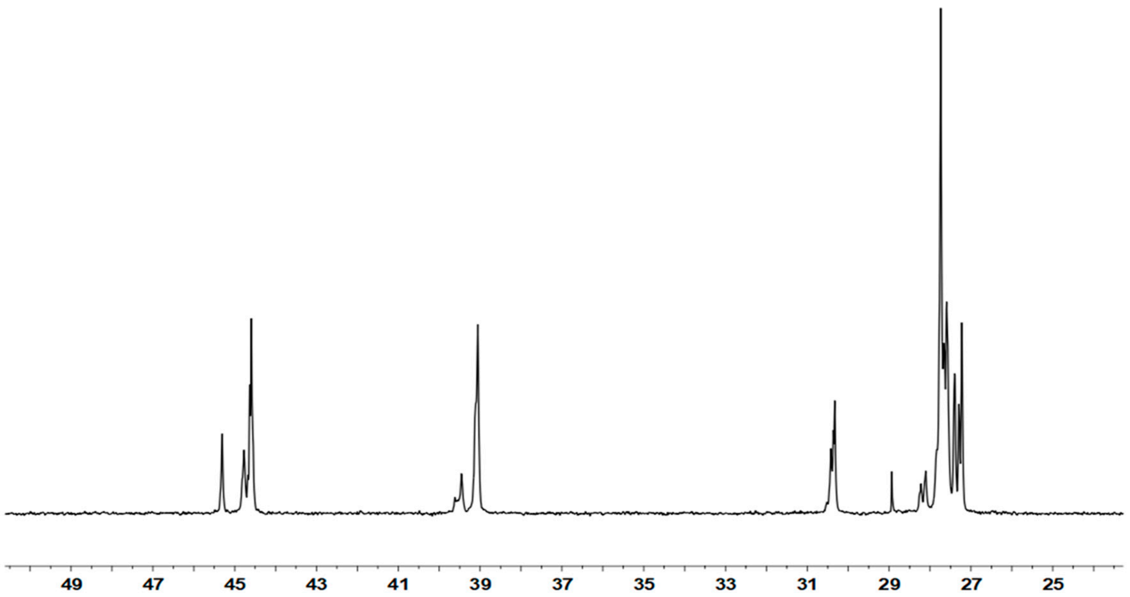

Figure 3S. The ^{13}C NMR spectrum of E-NB copolymer with (3) C_{NB}= 27.5 mol% obtained by **C1** complex (item according to Table 1)

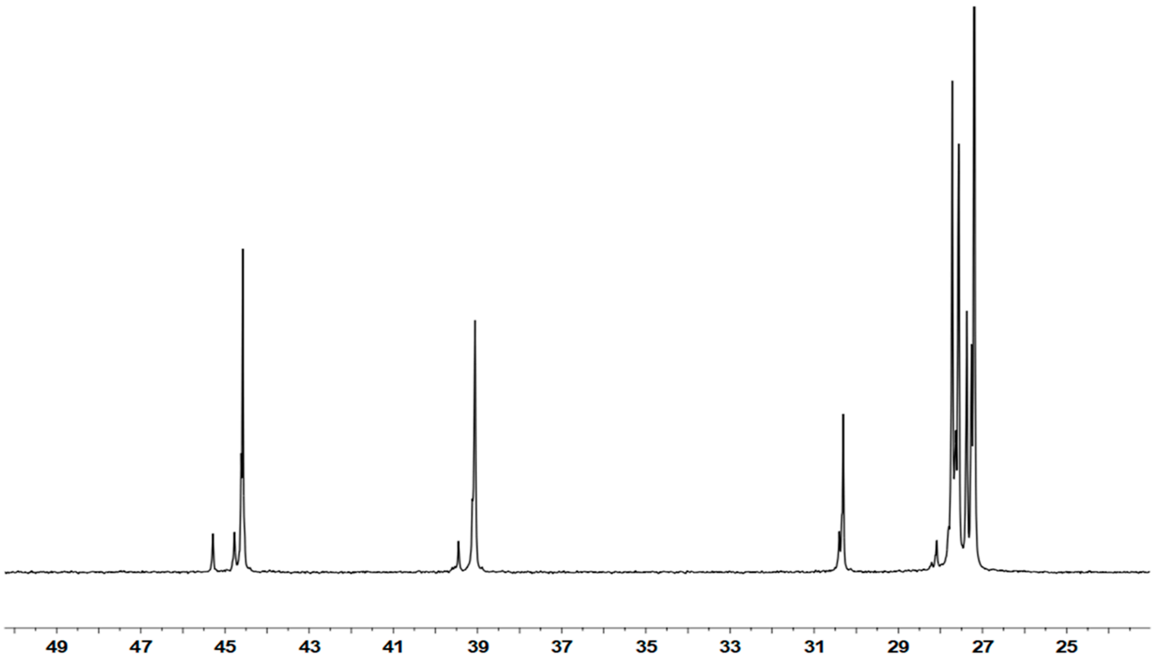

Figure 4S. The ^{13}C NMR spectrum of E-NB copolymer with (4) C_{NB}= 17.1 mol% obtained by **C2** complex (item according to Table 1)

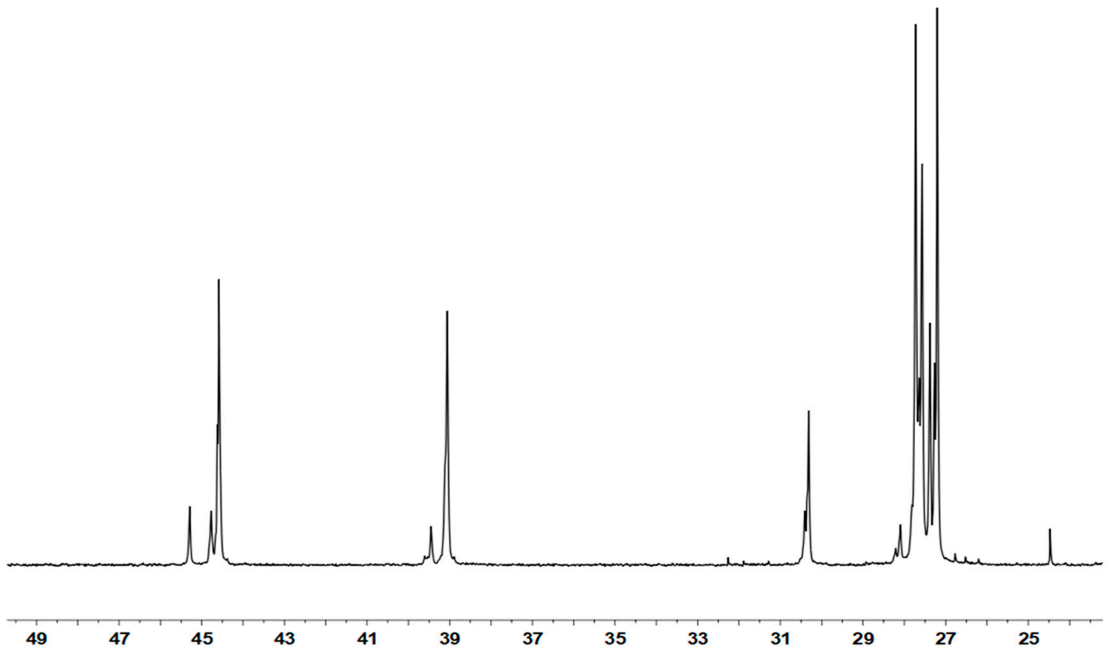

Figure 5S. The ^{13}C NMR spectrum of E-NB copolymer with (5) C_{NB}= 20.7 mol% obtained by **C2** complex (item according to Table 1)

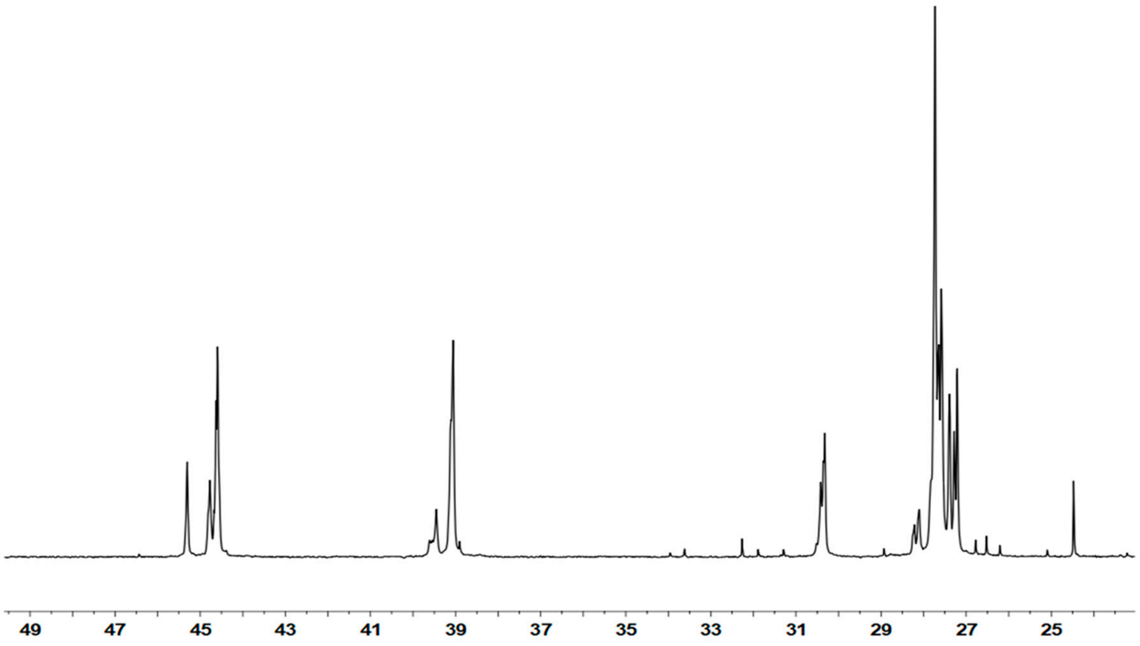

Figure 6S. The ^{13}C NMR spectrum of E-NB copolymer with (6) C_{NB}= 27.2 mol% obtained by **C2** complex (item according to Table 1)

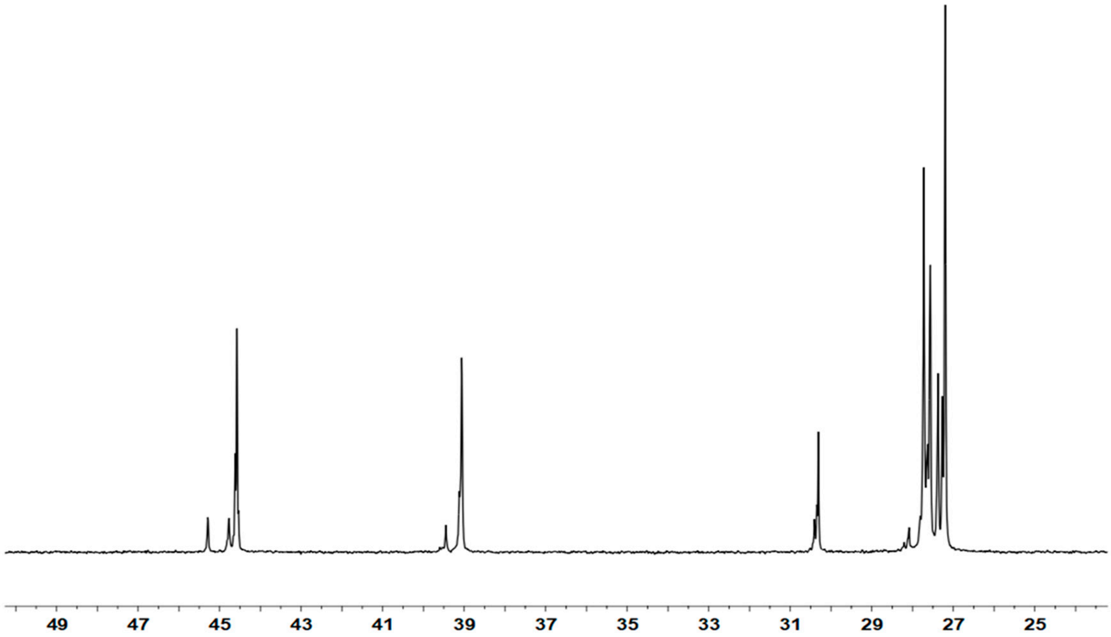

Figure 7S. The ^{13}C NMR spectrum of E-NB copolymer with (7) C_{NB}= 19.4 mol% obtained by **C3** complex (item according to Table 1)

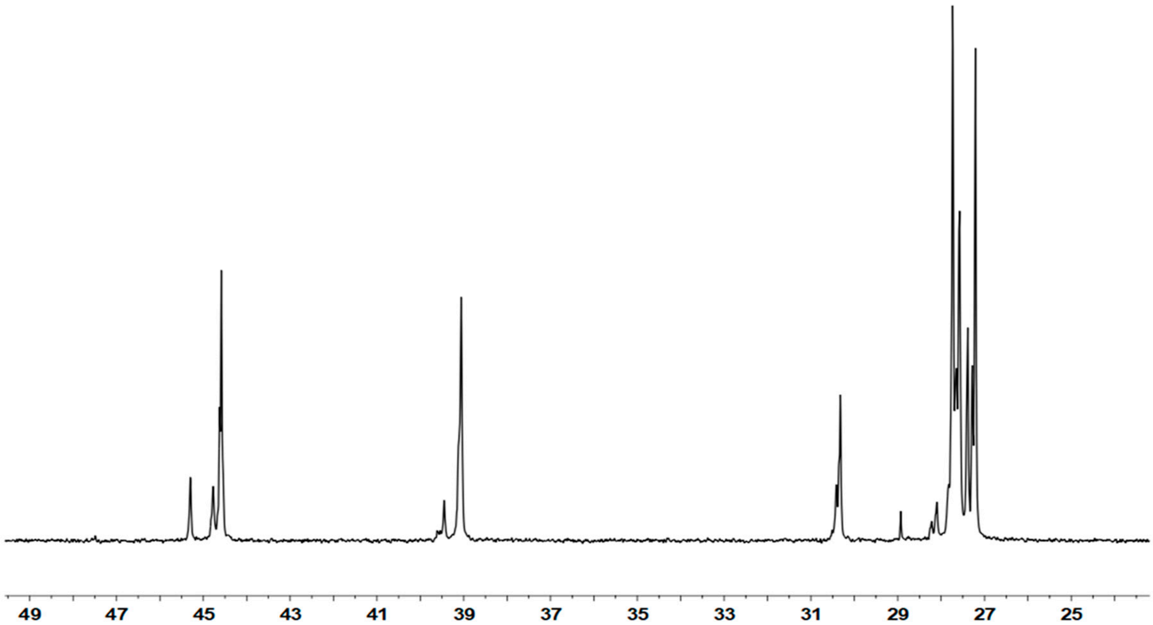

Figure 8S. The ^{13}C NMR spectrum of E-NB copolymer with (8) C_{NB}= 26.5 mol% obtained by **C3** complex (item according to Table 1)

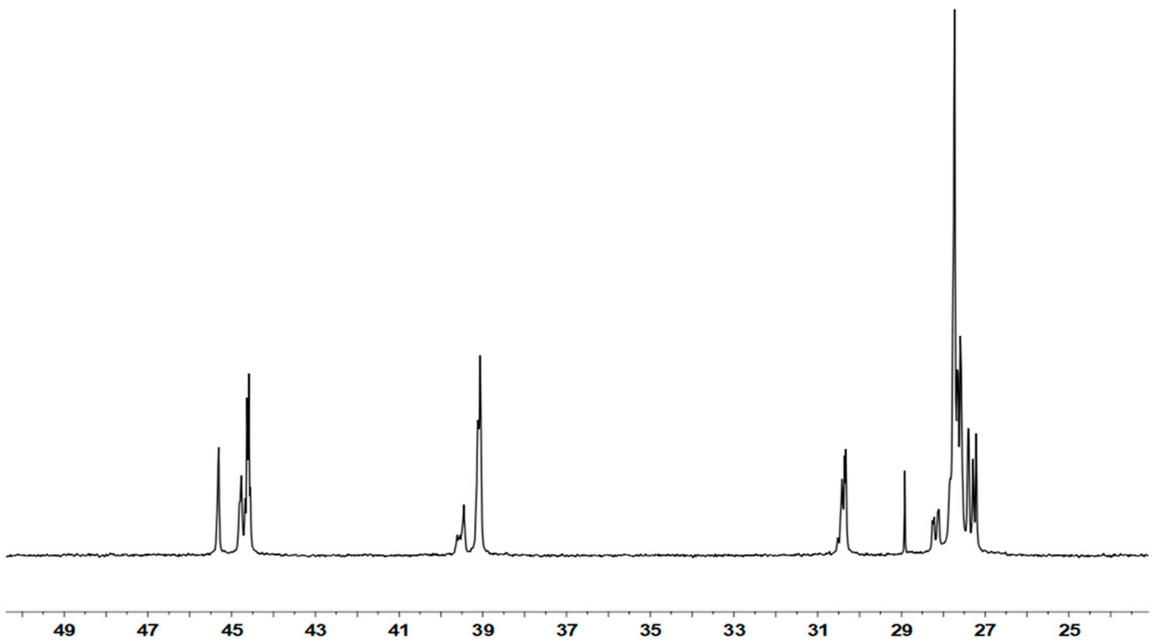

Figure 9S. The ^{13}C NMR spectrum of E-NB copolymer with (9) C_{NB}= 30.1 mol% obtained by **C3** complex (item according to Table 1)

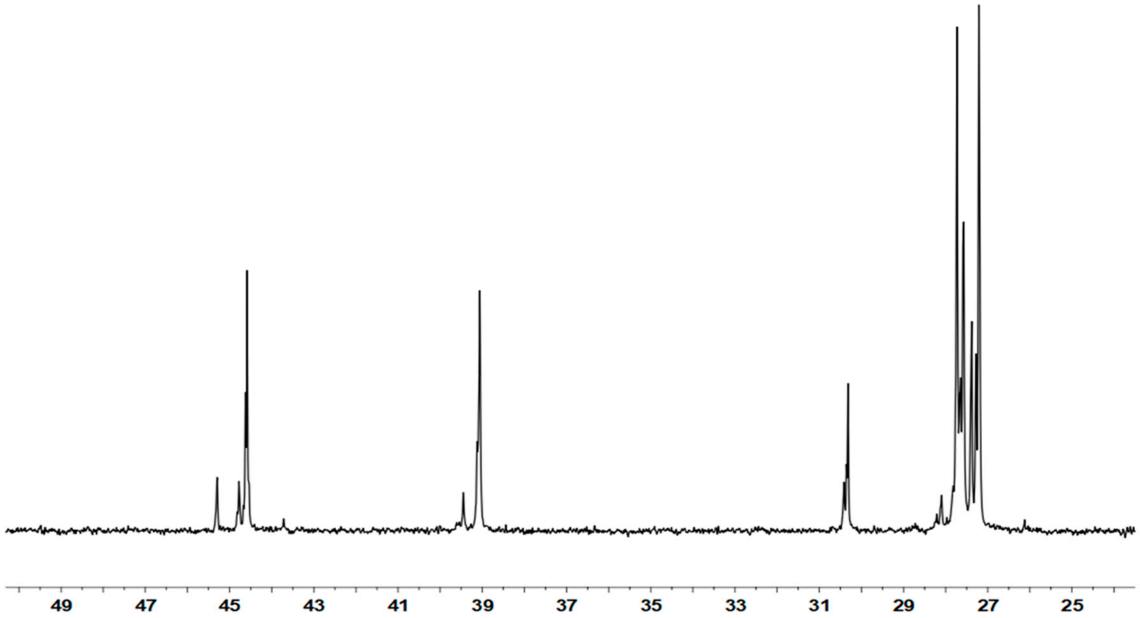

Figure 10S. The ^{13}C NMR spectrum of E-NB copolymer with (10) C_{NB}= 15.5 mol% obtained by **C4** complex (item according to Table 1)

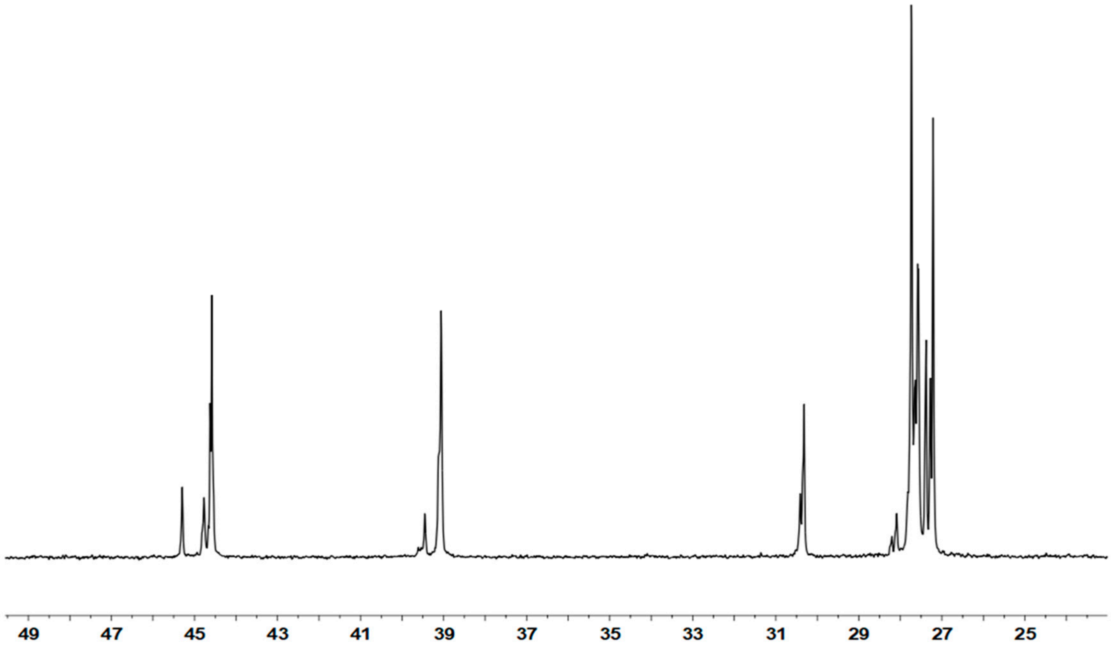

Figure 11S. The ^{13}C NMR spectrum of E-NB copolymer with (11) C_{NB}= 23.0 mol% obtained by **C4** complex (item according to Table 1)

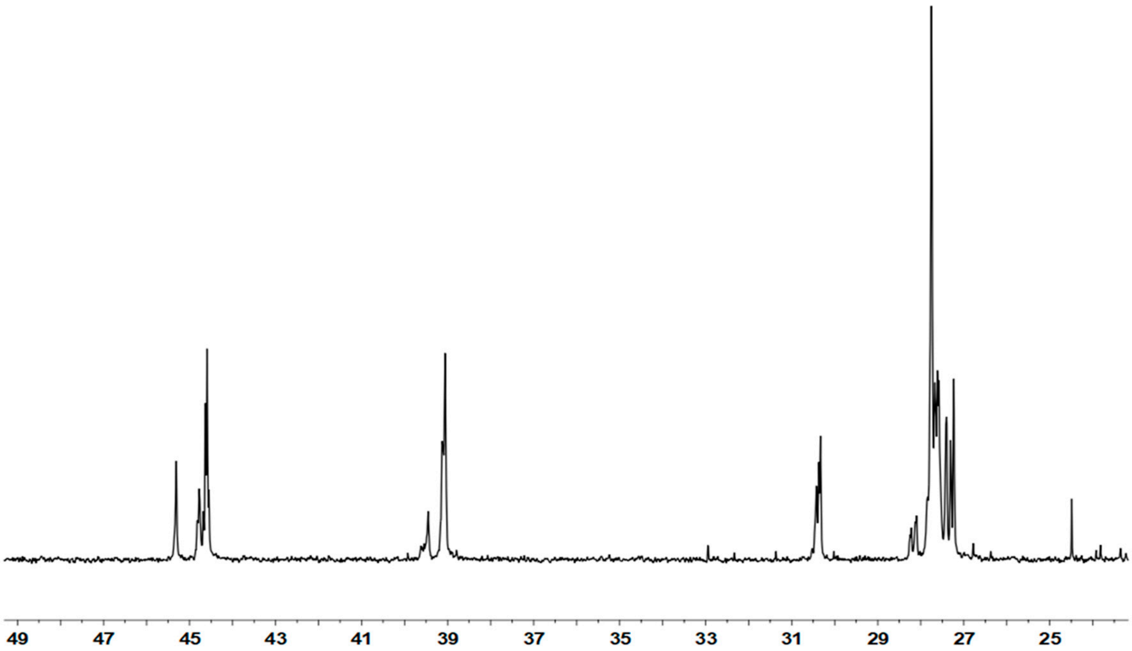

Figure 12S. The ^{13}C NMR spectrum of E-NB copolymer with (12) C_{NB}= 31.8 mol% obtained by **C4** complex (item according to Table 1)

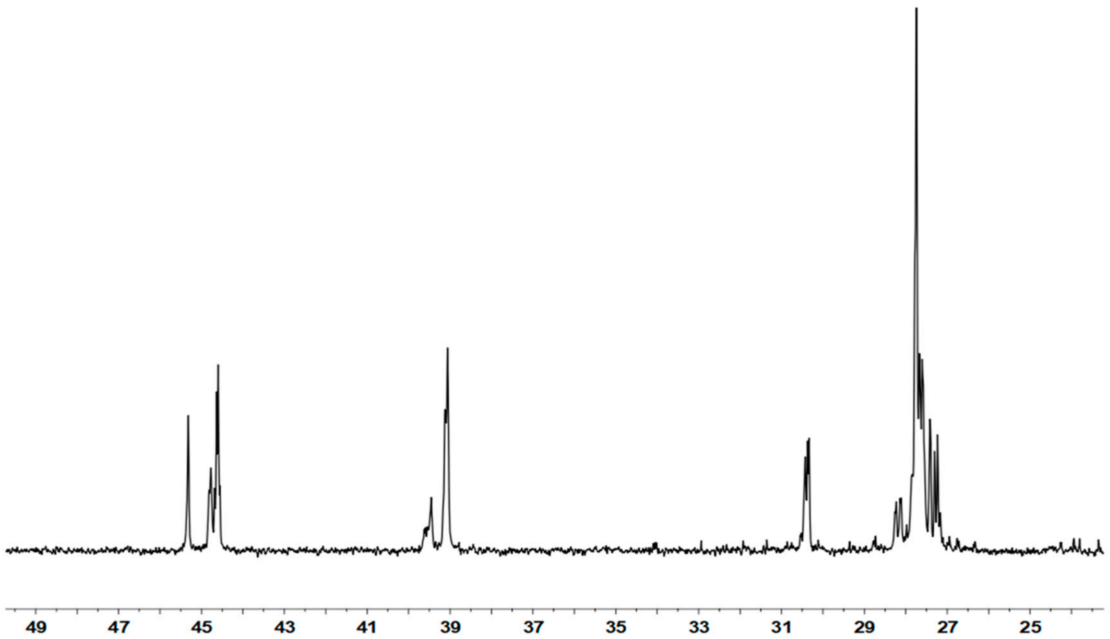

Figure 13S. The ^{13}C NMR spectrum of E-NB copolymer with (13) C_{NB}= 18.5 mol% obtained by **SIL/C4** complex (item according to Table 1)

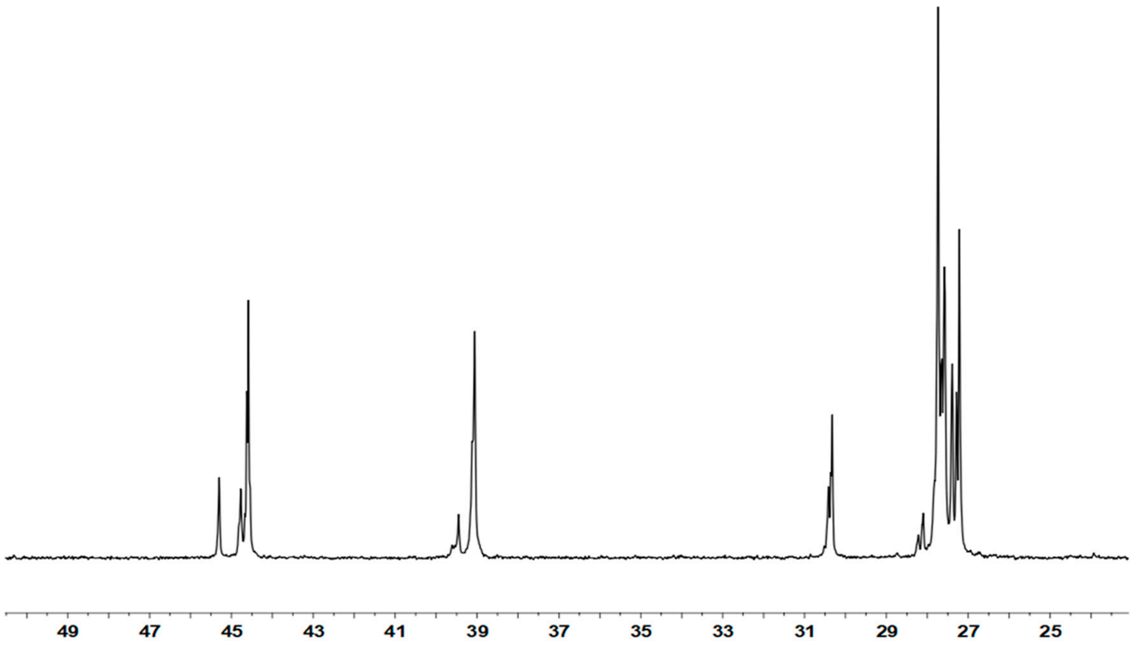

Figure 14S. The ^{13}C NMR spectrum of E-NB copolymer with (14) C_{NB}= 25.2 mol% obtained by **SIL/C4** complex (item according to Table 1)

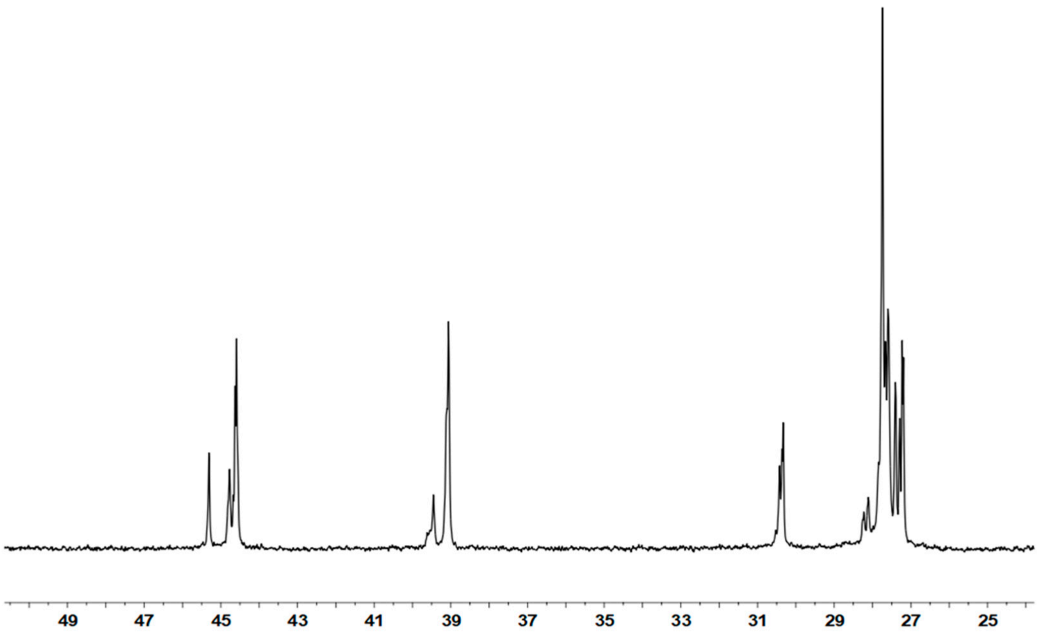

Figure 15S. The ^{13}C NMR spectrum of E-NB copolymer with (15) C_{NB}= 31.6 mol% obtained by **SIL/C4** complex (item according to Table 1)

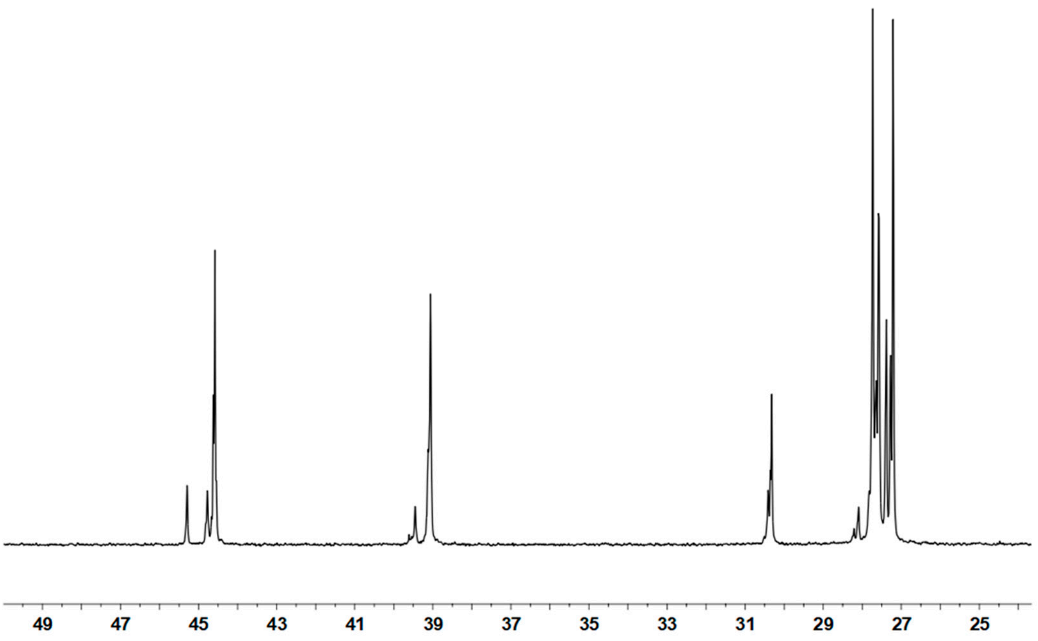

Figure 16S. The ^{13}C NMR spectrum of E-NB copolymer with (16) C_{NB}= 14.4 mol% obtained by **C5** complex (item according to Table 1)

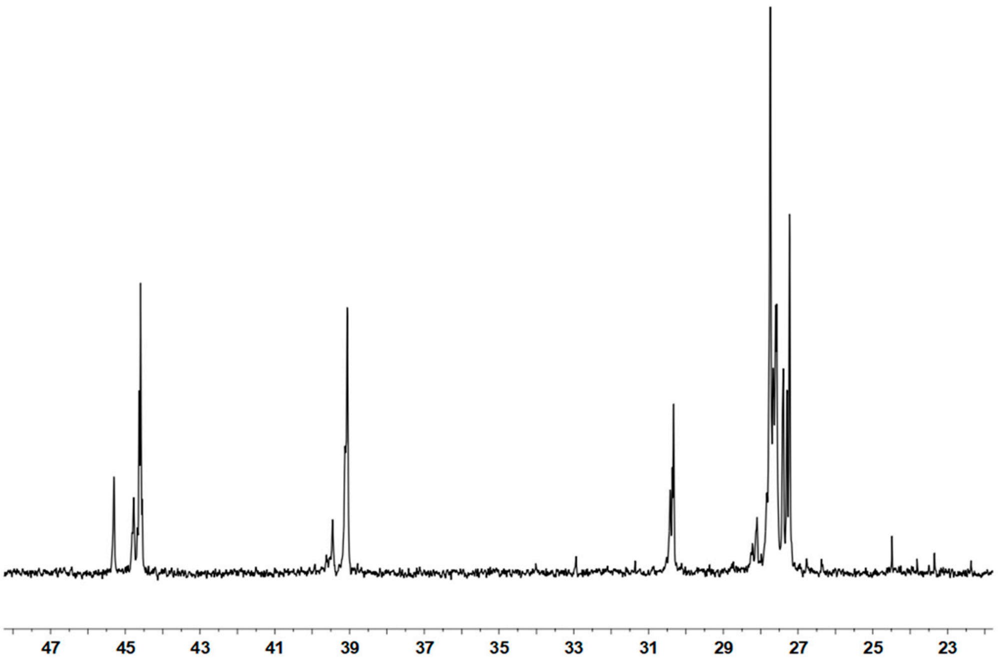

Figure 17S. The ^{13}C NMR spectrum of E-NB copolymer with (17) C_{NB}= 21.9 mol% obtained by **C5** complex (item according to Table 1)

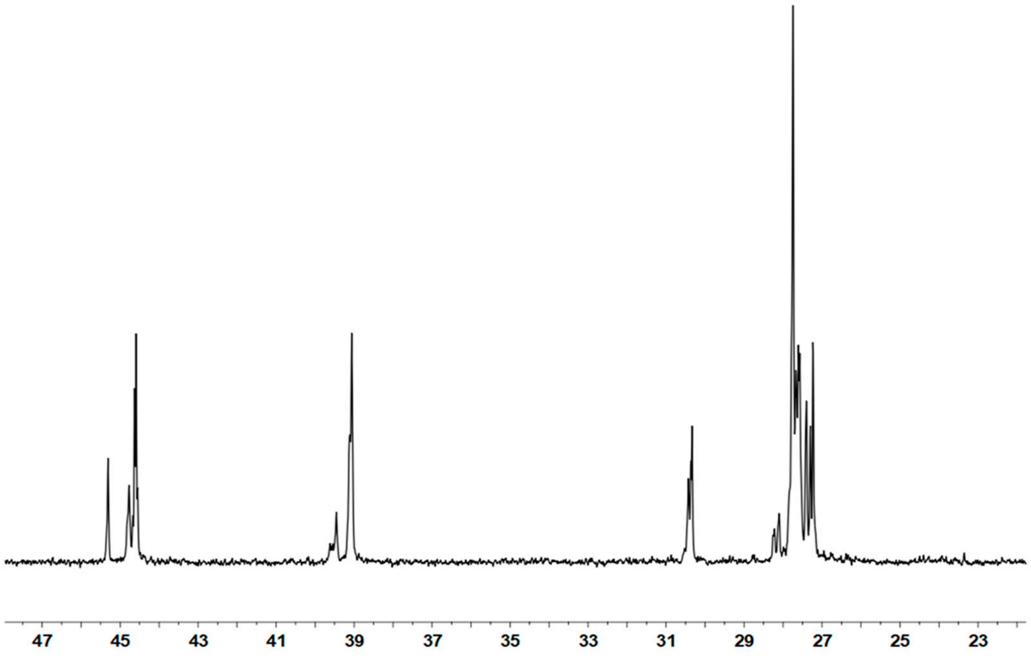

Figure 18S. The ^{13}C NMR spectrum of E-NB copolymer with (18) C_{NB}= 30.0 mol% obtained by **C5** complex (item according to Table 1)

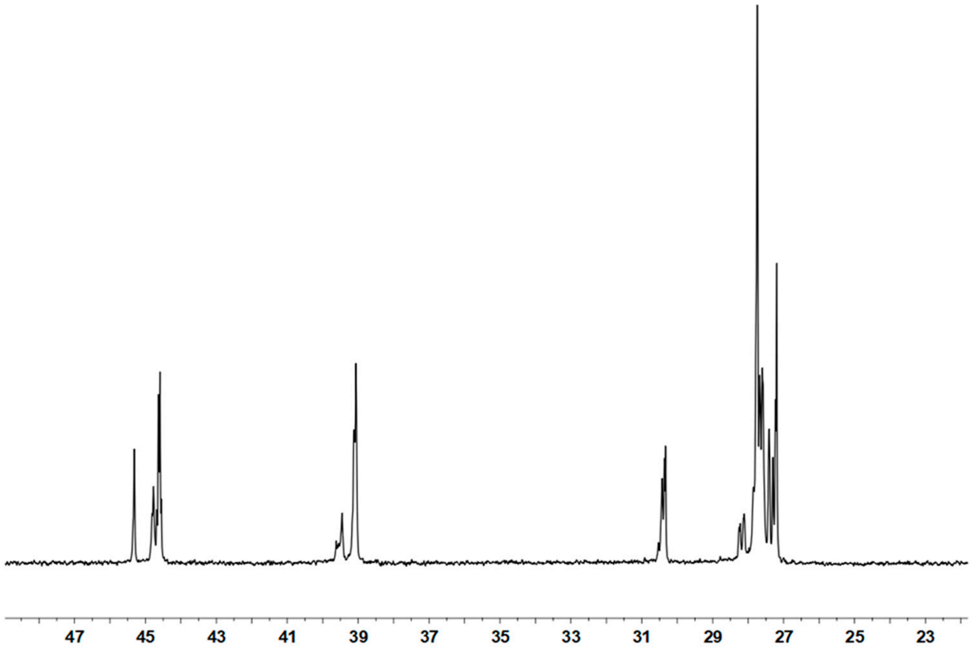

Figure 19S. The ^{13}C NMR spectrum of E-NB copolymer with (19) C_{NB}= 18.9 mol% obtained by **SIL/C5** complex (item according to Table 1)

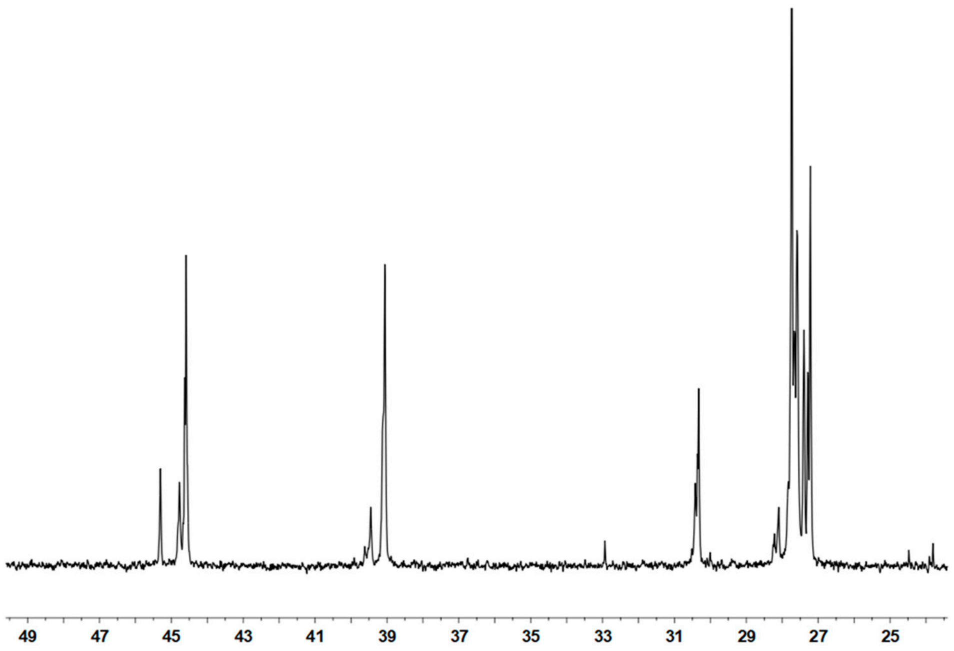

Figure 20S. The ^{13}C NMR spectrum of E-NB copolymer with (20) C_{NB}= 26.8 mol% obtained by **SIL/C5** complex (item according to Table 1)

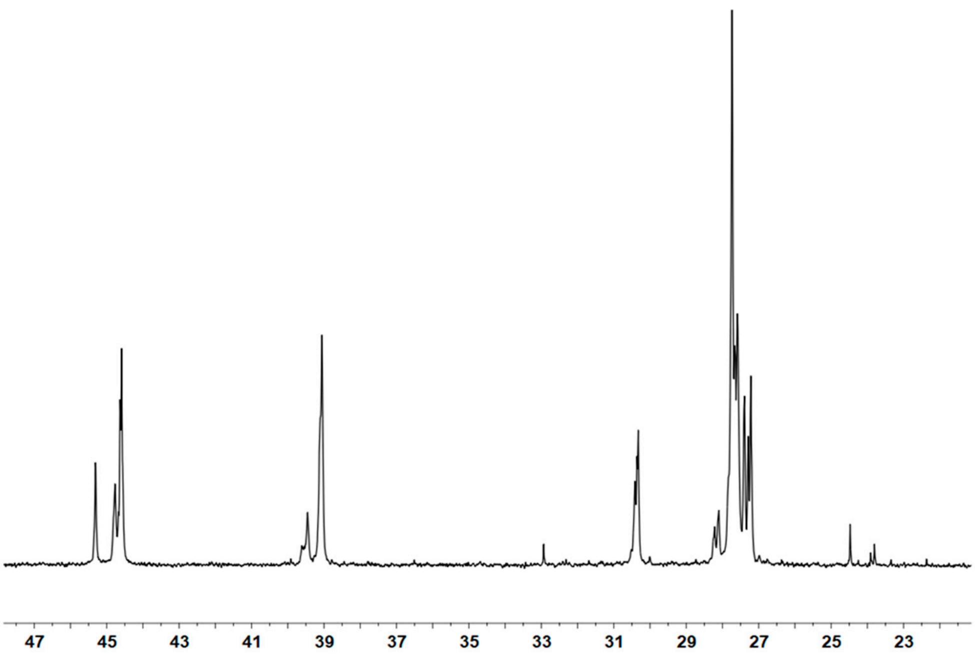

Figure 21S. The ^{13}C NMR spectrum of E-NB copolymer with (21) C_{NB}= 34.7 mol% obtained by **SIL/C5** complex (item according to Table 1)

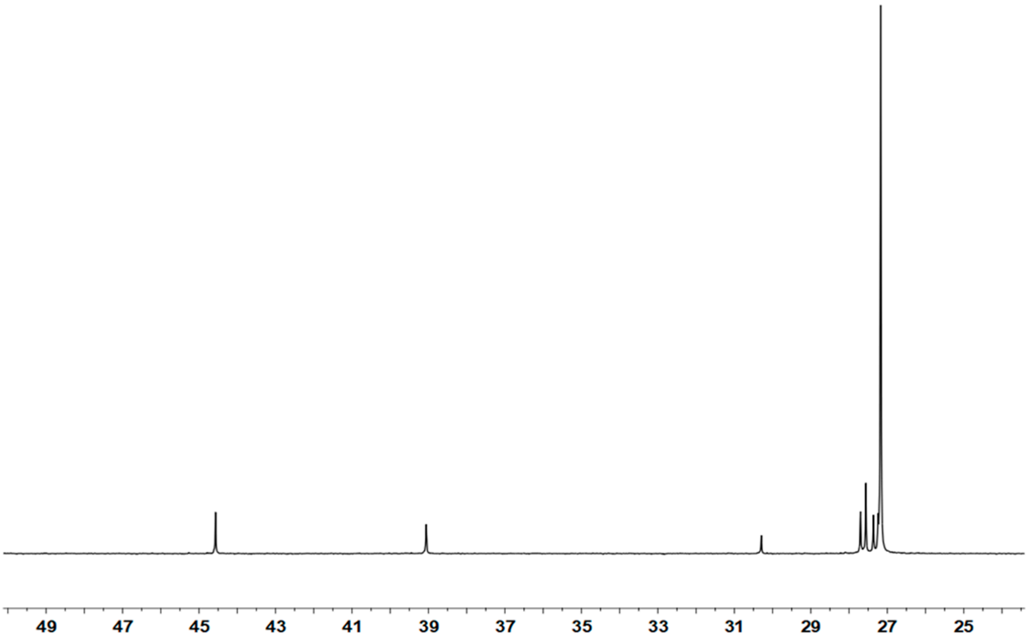

Figure 22S. The ^{13}C NMR spectrum of E-NB copolymer with (29) C_{NB}= 4.3 mol% obtained by **C6** complex (item according to Table 1)

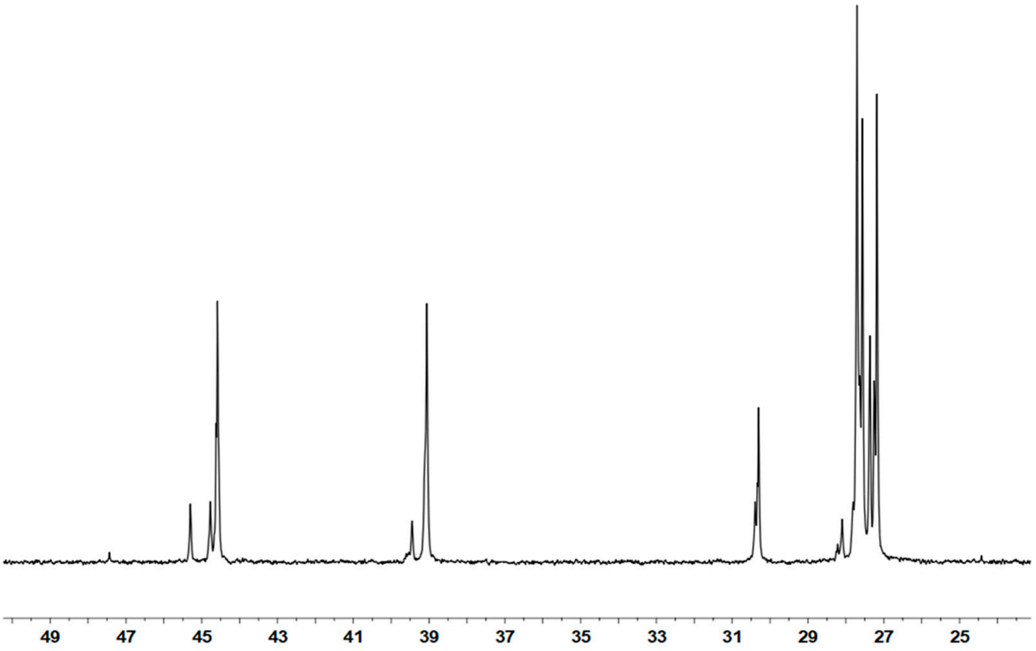

Figure 23S. The ^{13}C NMR spectrum of E-NB copolymer with (30) C_{NB}= 23.6 mol% obtained by **C6** complex (item according to Table 1)

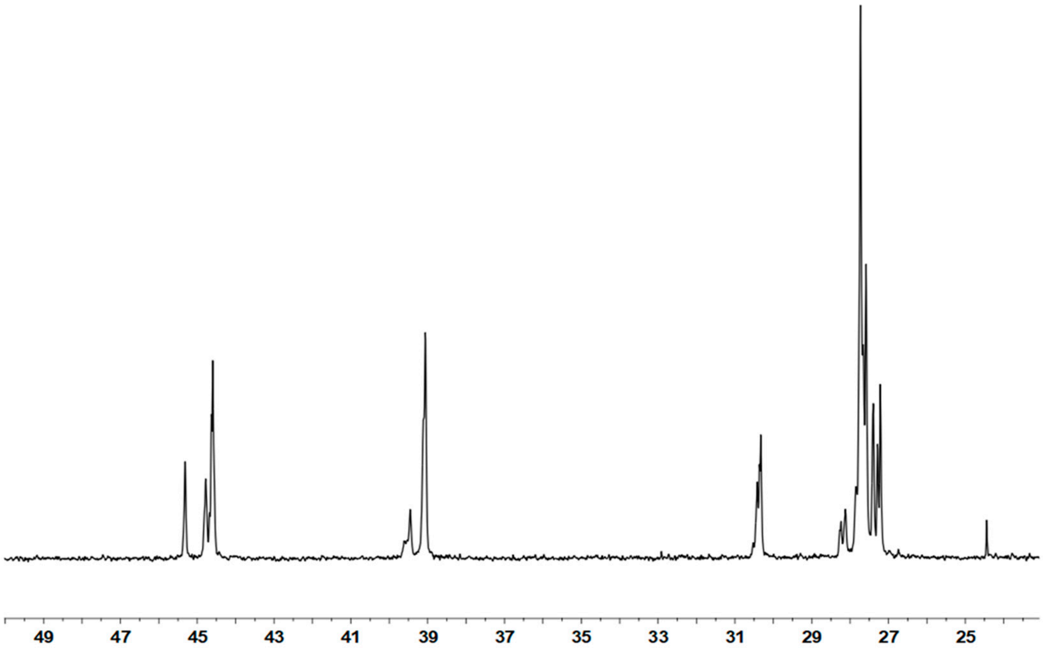

Figure 24S. The ^{13}C NMR spectrum of E-NB copolymer with (31) C_{NB}= 32.9 mol% obtained by **C6** complex (item according to Table 1)

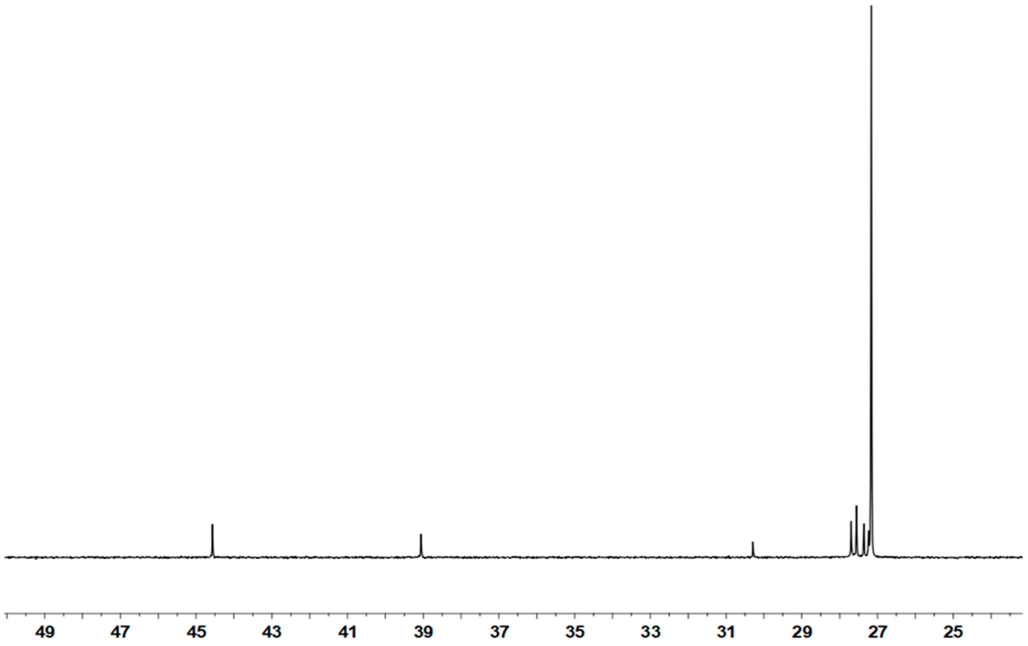

Figure 25S. The ^{13}C NMR spectrum of E-NB copolymer with (32) C_{NB}= 5.7 mol% obtained by **SIL/C6** complex (item according to Table 1)

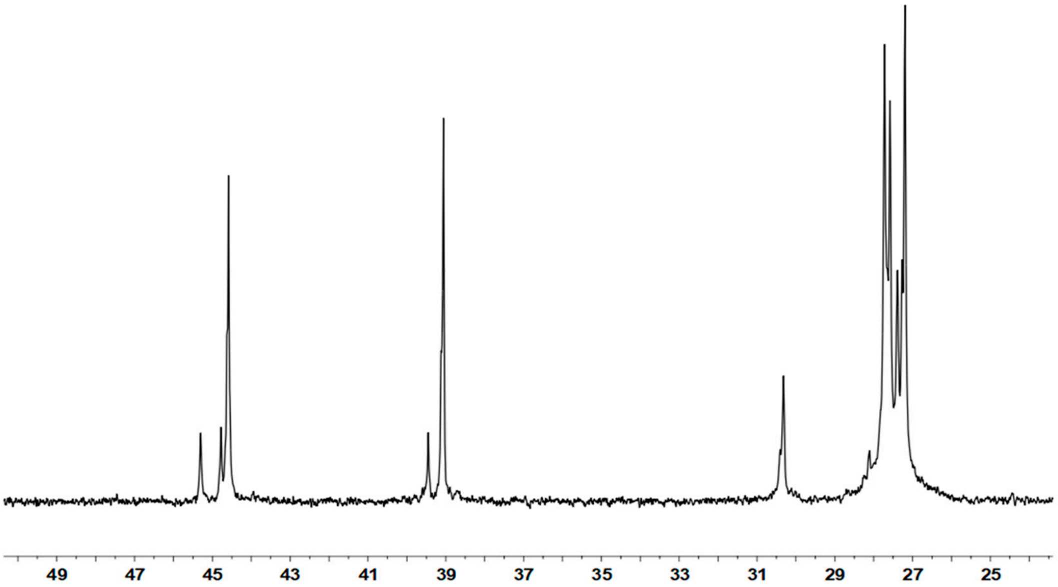

Figure 26S. The ^{13}C NMR spectrum of E-NB copolymer with (33) C_{NB}= 26.1 mol% obtained by **SIL/C6** complex (item according to Table 1)

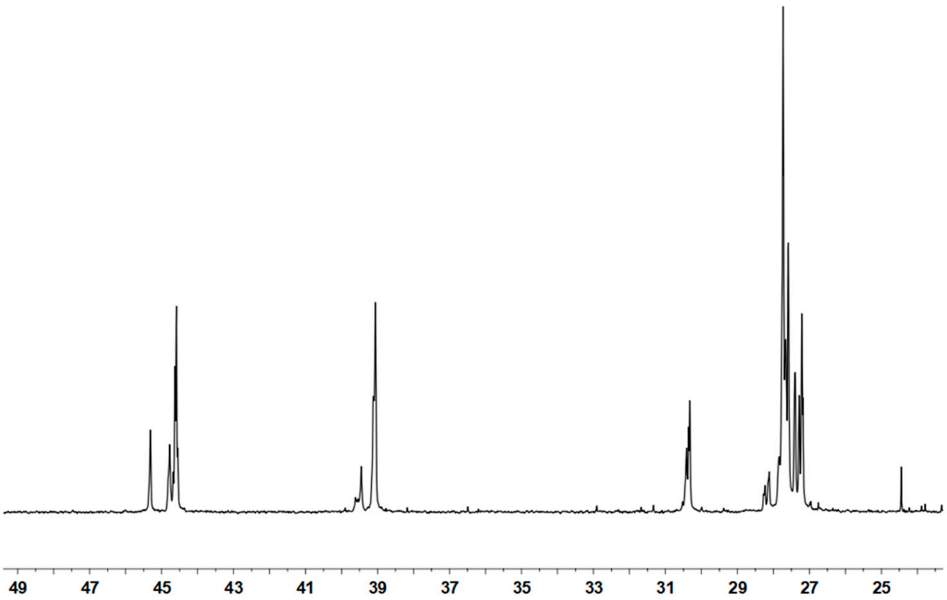

Figure 27S. The ^{13}C NMR spectrum of E-NB copolymer with (34) C_{NB}= 35.3 mol% obtained by **SIL/C6** complex (item according to Table 1)

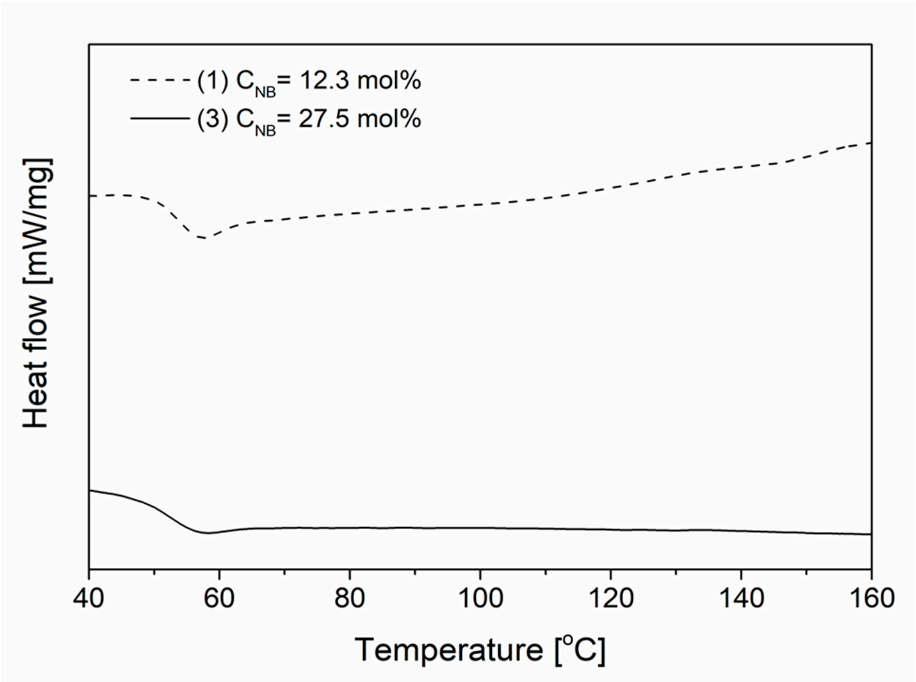

Figure 28S. The endothermic curves of E-NB copolymers obtained by **C1** complex (items according to Table 1)

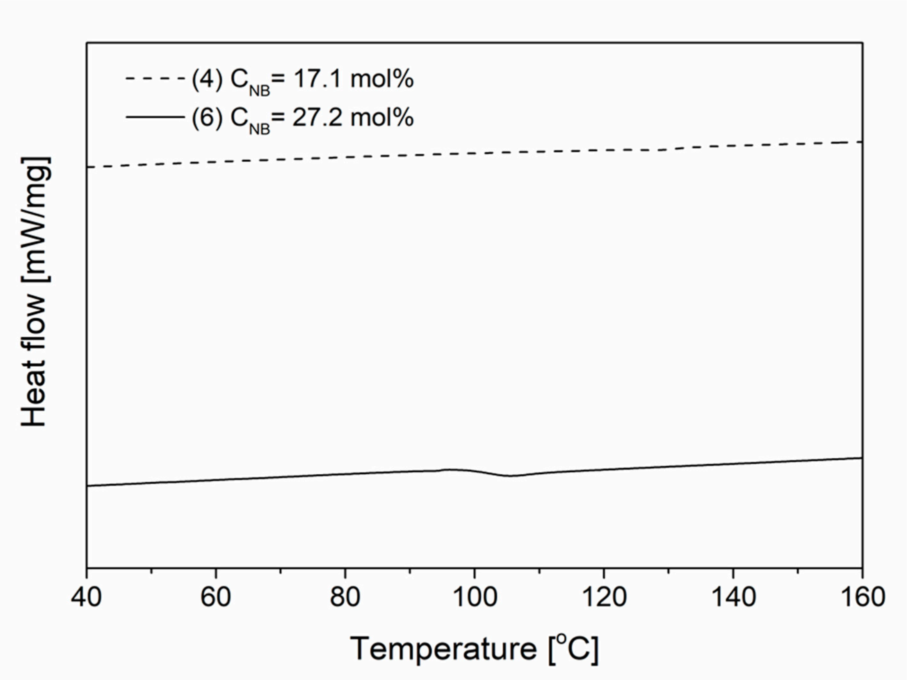

Figure 29S. The endothermic curves of E-NB copolymers obtained by **C2** complex (items according to Table 1)

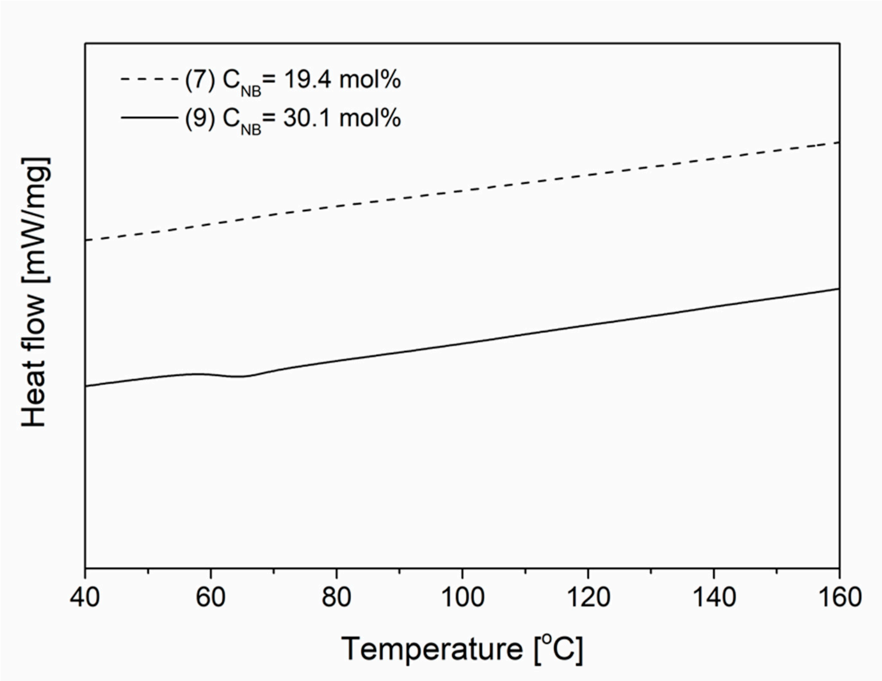

Figure 30S. The endothermic curves of E-NB copolymers obtained by **C3** complex (items according to Table 1)

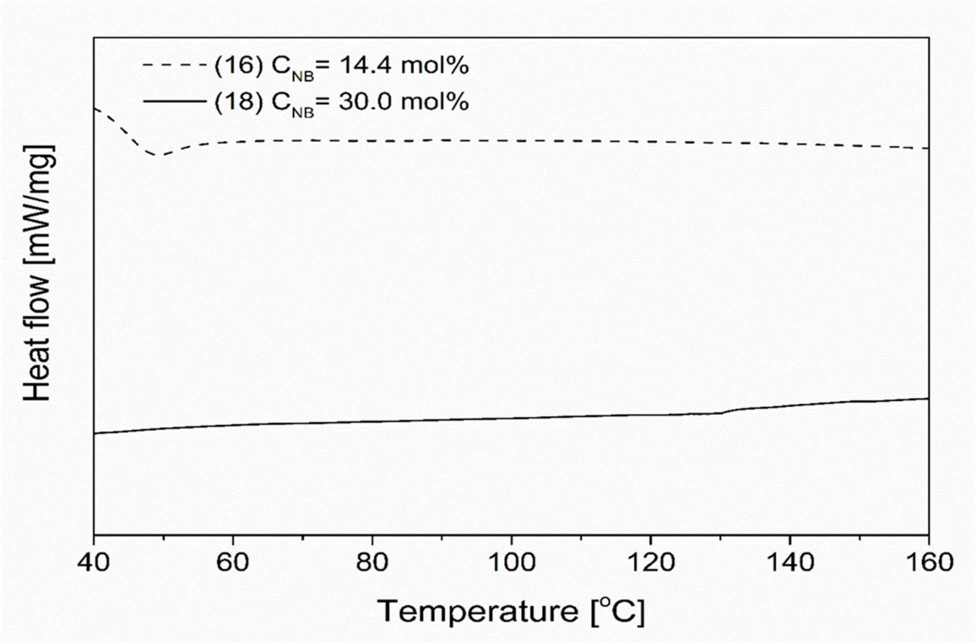

Figure 31S. The endothermic curves of E-NB copolymers obtained by **C5** complex (items according to Table 1)

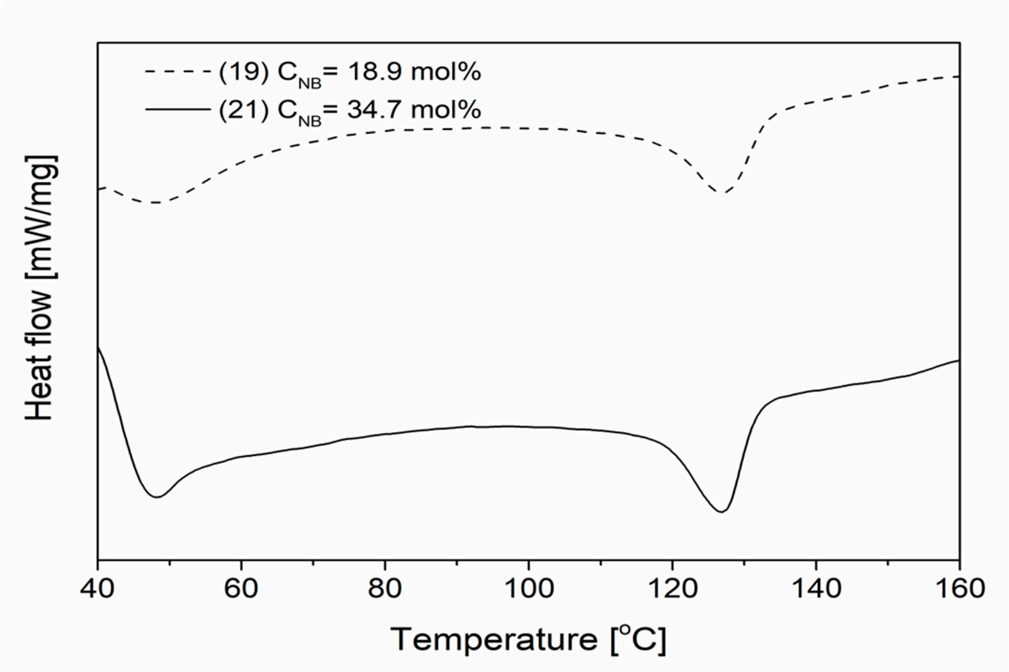

Figure 32S. The endothermic curves of E-NB copolymers obtained by **SIL/C5** complex (items according to Table 1)

The results of the determination of the structural parameters of copolymers (Table 1S) show that for all the investigated cases, copolymers were synthesized with low comonomer content. This means that ethylene exhibits much higher activity compared to that of the comonomer. In such cases the known copolymer composition equation used in the Fineman–Ross method can be replaced with the following simplified one:

$$m_{NB} m_e = r_e \times M_{NB} M_e \qquad (1)$$

where M_E and M_{NB} are, respectively, the concentrations of ethylene and norbornene in the reaction feed. In turn, m_{NB} and m_{NB} are content of ethylene and norbornene in the copolymer, respectively, and r_e is the reactivity ratio of ethylene.

Table S1. Structural parameters characterizing the ethylene/norbornene copolymers

Item	Catalyst	M_e [mol/dm^3]	M_{NB} [mol/dm^3]	m_e [mol%]	m_{NB} [mol%]	r_e	R^2
1			0.5	87.7	12.3		
2	**C1**	0.488	1.0	77.9	22.1	8.6	0.9728
3			1.5	72.5	27.5		
4			0.5	82.9	17.1		
5	**C2**	0.488	1.0	79.3	20.7	12.2	0.9733
6			1.5	72.8	27.2		
7			0.5	80.6	19.4		
8	**C3**	0.488	1.0	73.5	26.5	10.8	0.9656
9			1.5	69.9	30.1		
10			0.5	84.5	15.5		
11	**C4**	0.488	1.0	77.0	23.0	7.2	0.9979
12			1.5	68.2	31.8		
13			0.5	81.5	18.5		
14	**SIL/C4**	0.488	1.0	74.8	25.2	8.7	0.9998
15			1.5	68.4	31.6		
16			0.5	85.6	14.4		
17	**C5**	0.488	1.0	77.5	21.9	7.9	0.9995
18			1.5	70.0	30.0		
19			0.5	81.1	18.9		
20	**SIL/C5**	0.488	1.0	73.2	26.8	6.9	1.0000
21			1.5	65.3	34.7		
22			0.1	95.7	4.3		
23	**C6**	0.488	1.0	76.4	23.6	6.5	0.9987
24			1.5	67.1	32.9		
25			0.1	94.3	5.7		
26	**SIL/C6**	0.488	1.0	73.9	26.1	6.0	0.9973
27			1.5	64.7	35.3		
